# Humans trade off search costs and accuracy in a combined visual search and perceptual task

**DOI:** 10.3758/s13414-022-02600-5

**Published:** 2022-11-30

**Authors:** Ilja Wagner, Dion Henare, Jan Tünnermann, Anna Schubö, Alexander C. Schütz

**Affiliations:** 1 Experimental and Biological Psychology, University of Marburg, Gutenbergstraße 18, 35039 Marburg, Germany; 2 Center for Mind, Brain and Behavior, Marburg, Germany

**Keywords:** Visual search, Eye movements: *Other, Decision making

## Abstract

To interact with one’s environment, relevant objects have to be selected as targets for saccadic eye movements. Previous studies have demonstrated that factors such as visual saliency and reward influence saccade target selection, and that humans can dynamically trade off these factors to maximize expected value during visual search. However, expected value in everyday situations not only depends on saliency and reward, but also on the required time to find objects, and the likelihood of a successful object-interaction after search. Here we studied whether search costs and the accuracy to discriminate an object feature can be traded off to maximize expected value. We designed a combined visual search and perceptual discrimination task, where participants chose whether to search for an easy- or difficult-to-discriminate target in search displays populated by distractors that shared features with either the easy or the difficult target. Participants received a monetary reward for correct discriminations and were given limited time to complete as many trials as they could. We found that participants considered their discrimination performance and the search costs when choosing targets and, by this, maximized expected value. However, the accumulated reward was constrained by noise in both the choice of which target to search for, and which elements to fixate during search. We conclude that humans take into account the prospective search time and the likelihood of successful a object-interaction, when deciding what to search for. However, search performance is constrained by noise in decisions about what to search for and how to search for it.

**Supplementary Information:**

The online version contains supplementary material available at 10.3758/s13414-022-02600-5.

## Introduction

Humans have foveated visual systems, which are characterized by a functional division of the eye’s retina in a photoreceptor-dense foveal part, used for high-acuity perception, and a peripheral part, where visual acuity gradually decreases and susceptibility to clutter increases with rising eccentricity (for reviews, see Rosenholtz, 2016; Stewart et al., 2020; Strasburger et al., 2011). To overcome these processing differences across the retina, humans reorient their fovea to different points of interest in visual scenes, using saccadic eye movements. Necessarily, each saccade results from a decision about which part of the world to select as a saccade target, as well as a target for high-acuity perception, and which parts to ignore.

The factors that influence decisions about target selection for saccades have been studied extensively (for reviews, see Hayhoe, 2017; Schütz et al., 2011; Tatler et al., 2011). For example, previous research showed that low-level features of scenes, such as visual saliency (Itti & Koch, 2000; Kümmerer et al., 2016), motivational aspects of stimuli, such as a monetary reward linked to saccade targets (Liston & Stone, 2008), and the prospective gain in task-relevant visual information after saccades (for reviews, see Gottlieb, 2012, 2018; Gottlieb et al., 2014; Gottlieb & Oudeyer, 2018) influence saccade target selection. For the information gain of saccades, some studies found evidence that the human oculomotor system is not only sensitive to this factor, but it also optimizes saccade target selection to maximize information gain (Eckstein et al., 2015; Hoppe & Rothkopf, 2019; Najemnik & Geisler, 2005, 2008; Paulun et al., 2015; Peterson & Eckstein, 2012; Renninger et al., 2007; Yang et al., 2016). However, other studies demonstrated that this is not always the case (Ackermann & Landy, 2013; Araujo et al., 2001; Clarke et al., 2022b; Clarke & Hunt, 2016; Ghahghaei & Verghese, 2015; Morvan & Maloney, 2012; Nowakowska et al., 2017, 2021; Tsank & Eckstein, 2017; Verghese, 2012; Wolf et al., 2019; Zhou & Yu, 2021). For instance, human eye-movement behavior during visual search can be explained equally well by a stochastic model (Clarke et al., 2016), or better by a model that focuses on minimizing costs of saccades, while maintaining a sufficiently high task performance (Zhou & Yu, 2021). For more complex testing environments with multiple competing sources of information, some studies demonstrated that humans can combine information about saliency and reward for saccade target selection (Navalpakkam et al., 2010; Schütz et al., 2012; Stritzke et al., 2009): Navalpakkam et al. (2010), for example, showed that participants can, in accordance with predictions of an ideal Bayesian observer, dynamically trade off saliency and reward in visual search displays to select saccade targets that maximize expected value.

However, in cluttered real-world environments, populated by an abundance of objects, different potential saccade targets might have equal saliency and comparable motivational value (e.g., two equally favorable products with similarly conspicuous packaging on supermarket shelves). Furthermore, a single saccade, which in the paradigm of Navalpakkam et al. (2010) was in most cases sufficient to find reward-maximizing targets, is typically not enough to locate sought-after objects outside the laboratory. Instead, time-consuming visual search (for reviews, see Eckstein, 2011; Lleras et al., 2022; Nakayama & Martini, 2011; Wolfe, 2021) is often required, where parts of scenes have to be fixated sequentially, in order to locate relevant targets among irrelevant distractors (e.g., finding one’s favorite product amidst products with similar looking packaging). Additionally, saccades in real-world environments are often not ends in themselves, but objects are typically chosen as saccade targets to be interacted with after eye movements (e.g., lifting one’s favorite product from a supermarket shelf after finding it) and those interactions might not always be successful (e.g., when the desired product is placed too high up in a shelf), especially when a searcher is under time pressure (e.g., shortly before closing-time). Finally, eye movements in natural environments typically do not yield monetary rewards, but provide visual information for a given task (Foley et al., 2017; for a review, see Gottlieb & Oudeyer, 2018; Horan et al., 2019; Paeye et al., 2016). Thus, in stimulus-filled environments with multiple comparably valuable and salient choice-alternatives, the quality of competing saccade targets might not primarily depend on their relative motivational value and saliency. Instead, saccade target quality depends on factors such as the necessary time to find sought-after objects under time pressure (i.e., their relative search costs) and the likelihood of successful interaction with them after search.

Previous research has shown that both the difficulty to discriminate targets from distractors and the perceptual discriminability of stimuli per se influence eye-movement strategies (Pomplun et al., 2013), as well as fixation durations (Becker, 2011; Näsänen et al., 2001; Williams & Pollatsek, 2007), during visual search. Another line of research demonstrated that humans take time constraints into account when making decisions about when to stop looking for targets during visual foraging (Cain et al., 2012; Ehinger & Wolfe, 2016) and how much time to spend on perceptual tasks more generally (Jarvstad et al., 2012).

For target selection, some studies demonstrated that when the ratio of two types of distractors, from which ones shares a feature with the target, is manipulated, human observers are more likely to fixate distractors from the smaller set during search (Egeth et al., 1984; Shen et al., 2000; Sobel & Cave, 2002), and this effect cannot be explained by saliency of the smaller set (Kaptein et al., 1995). Similarly, a series of recent studies showed that the time required to find targets amidst distractors also influences decisions about what target to search for. Irons and Leber (2016, 2018) showed their participants large search displays with 54 colored elements. However, instead of instructing participants to search for one fixed target in every trial, the authors presented two differently-colored targets in each trial, and participants could freely choose which target they want to search for. To manipulate the efficiency of either searching for one or the other target, Irons and Leber (2016, 2018) varied the proportion of distractors in search displays which had a similar color as one or the other target options. As a consequence, search efficiency (i.e., how many distractors need to be inspected before finding a target) depended on the number of similar looking distractors in a display, and participants were hypothesized to dynamically update which target they search for, depending on the number of similar looking distractors in search displays. The authors found large individual differences in their data, with half of their participants failing to maximize task-performance. However, a subset of participants, although showing unnecessary target switches and delays in target switches, had a tendency to spontaneously choose targets that took less time to find, and to fixate distractors that shared some of their chosen target’s features (see also Bergmann et al., 2020). The observed individual strategies of participants were found to be stable over time as well as context-specific (Clarke et al., 2020; Irons & Leber, 2018), and participants’ performance improved when previews of search displays were shown before trials (Hansen et al., 2019).

Although the prospective search costs of target options seem to influence search behavior of some participants, it is currently unknown if other factors, such as the relative difficulty of interacting with targets (e.g., discriminating them for a perceptual task), have a similar influence on target selection during visual search. Furthermore, it is currently unknown if discrimination accuracy can be weighed against the prospective search costs of competing target-options to optimize eye-movement behavior. Here we studied whether human participants can, under time-pressure, dynamically trade off the prospective search costs as well as discrimination accuracy of two competing targets to maximize expected value during visual search.

## Methods

### Participants

We recorded data from 21 participants. Three participants were undergraduate student assistants in the laboratory, whereas the rest of the sample consisted of naïve undergraduate students. Although student assistants were familiar with eye tracking, they were naïve with respect to the paradigm, and the hypothesis of the present experiment. One participant had to be excluded after participating in the single-target condition, because he/she did not comply with the instructions. A second participant was excluded because of extreme average search times in both conditions (single-target: 7,874 ms; remaining participants: *M* = 2,546 ms, 95% confidence interval (CI_95%_) = [2,228 ms, 2,865 ms]; double-target: 6,107 ms; remaining participants: *M* = 2,320 ms, CI_95%_ = [2,016 ms, 2,625 ms) that led to a comparatively small number of completed trials (single-target: 51 trials; double-target: 64 trials). However, the general pattern of our results did not change irrespective of whether data from this participant was included or not. The remaining 19 participants had a mean age of 23 years (min.: 19 years, max.: 29 years, 15 female). Sample size was determined based on previous studies that focused on similar research questions (Clarke et al., 2016; Clarke et al., 2022b; Nowakowska et al., 2021; Zhou & Yu, 2021). Additionally, our study uses a computational model to quantify individual differences as its main analysis tool. This has been previously shown to be a powerful approach, even for studies with comparatively small sample sizes (Smith & Little, 2018).

All participants were naïve as to the purpose of the experiment and had normal or corrected-to-normal vision. Participants were compensated with 8 €/h and an additional performance-dependent bonus payout. The latter was determined by translating the sum of points, which participants acquired throughout both conditions of the experiment, into Euros, with one score point (defined as 0.01 points) corresponding to one Eurocent (e.g., a participant with a final score of 4.10 received a total bonus payout of 4.10€). The average total bonus payout was 3.69€ (min.: 0.02€, max.: 7.34€).

### Equipment

All experiments were conducted using the Psychtoolbox (Brainard, 1997) in Matlab R2016b (The MathWorks, Natick, MA, USA). Stimuli were presented on a back-projection setup, using a PROPixx projector (VPixx Technologies Inc., Saint-Bruno, Quebec, Canada) and a Stewart Filmscreen screen (Stewart Filmscreen Corporation, Torrance, California, USA). The screen had a size of 90.70 × 51.00 cm, a spatial resolution of 1,920 × 1,080 pixels and a refresh rate of 120 Hz. The viewing distance was 106 cm. Background color was set to gray (R: 128, G: 128, B: 128, luminance: 69.6 cd/m^2^) and the screen was calibrated to ensure linear gamma correction. A hotspot correction was used to ensure equal luminance across the screen. Eye movements of the right eye were recorded with an EyeLink 1000+ (SR Research Ltd., Ontario, Canada) at a sampling rate of 1,000 Hz. The Eyelink Toolbox was used to control the eye tracker (Cornelissen et al., 2002).

### Stimuli

**Fig. 1:**
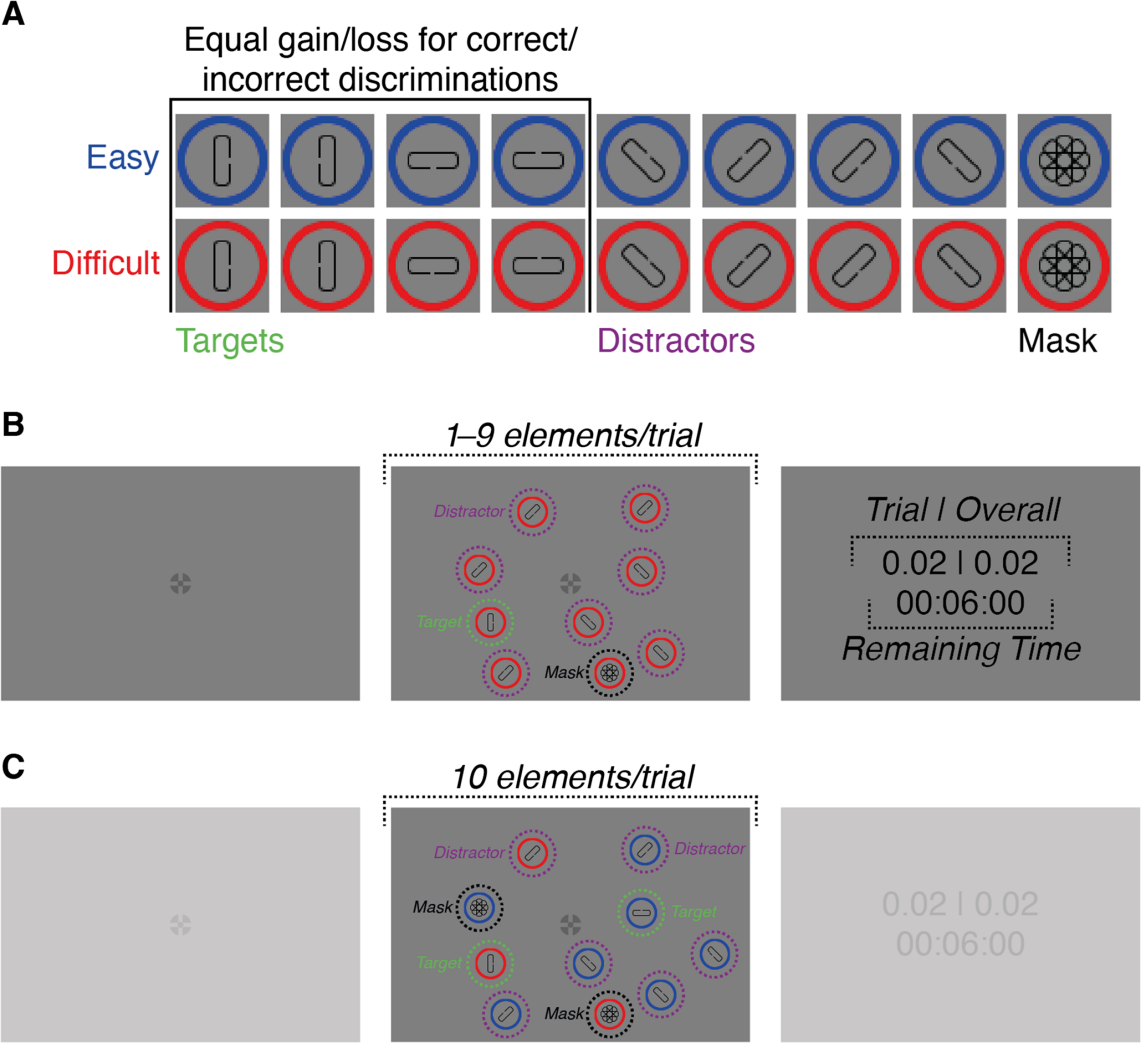
Stimuli and trial procedure. (**A**) Stimuli were differently oriented rectangles, centered within blue or red circles. The stimulus pool was divided into two sets: the easy and difficult set. Each set consisted of one target (rectangles oriented horizontally or vertically), several distractor variations (oriented diagonally), and a mask stimulus (rectangles of all orientations superimposed onto each other). Elements of both sets differed in their ring color, and the size of a gap located on one of the two long sides of their rectangles. Depending on gap size, targets were either difficult (small gap) or easy (large gap) to discriminate. (**B**) In the single-target condition, participants were instructed to find a target, presented among 0–8 distractors from the target’s set, and to discriminate the position of the gap. The number of distractors, and the set that was shown in a particular trial, were both varied across trials. Participants were given 6 min and 30 s to complete as many trials as they could. (**C**) In the double-target condition, participants had the same task as in the single-target condition, however, here, both targets and eight distractors, composed of elements from both sets, were shown in each trial. The relative number of easy and difficult distractors was varied across trials. (**B**–**C**) Although trial duration was unlimited, viewing time of individual stimuli was limited: Each stimulus could be viewed for up to 500 ms, after which it was replaced by a mask. Differences between conditions are highlighted. Stimuli are not drawn to scale. Dashed lines/circles and italic text were not part of the display and are shown for illustration purposes only. (**A**–**C**) For illustration purposes, rectangles within circles are drawn in black, whereas they were drawn in gray during the experiment

A combination of cross and bull’s eye (total diameter: 0.60°) was used as fixation cross (Thaler et al., 2013). Custom stimuli were used as targets, distractors, and mask stimuli (Fig. 1A). All stimuli were made up of an unfilled circle with a colored outline (red (R: 255, G: 0, B: 0, luminance: 43.70 cd/m^2^) or blue (R: 0, G: 0, B: 255, luminance: 6.10 cd/m^2^), circle diameter: 1.20°, line thickness of outline: 0.10°) and a gray (R: 109, G: 109, B: 109, luminance: 59.70 cd/m^2^) round-edged rectangle, centered within the circle (length longer side: 0.69°, length shorter side: 0.23°, line thickness of outline: 0.03°). For targets, the rectangle was oriented either horizontally (90° relative to vertical) or vertically (0°), whereas it had one of two possible diagonal orientations (45°, 135°) for distractors. For mask stimuli, rectangles of all orientations were superimposed onto each other.

Depending on the color of the circle outline, stimuli belonged to one of two sets: the easy and difficult set. For the easy set, the target and distractors had a comparatively large gap (0.08°) on one of its long sides (i.e., the gap was left or right if the rectangle was oriented vertically and up or down if it was oriented horizontally), whereas the gap was smaller for elements of the difficult set (0.05°). Gap sizes were chosen based on pilot data such that discriminating the gap location on the easy target during fixation yielded higher perceptual performance than discriminating the difficult target. Mask stimuli did not have a gap. Anti-aliasing was applied to both the circle outline and the rectangle within the circle. To avoid interference with the discrimination task, anti-aliasing on targets was only applied to rounded edges, but not the long rectangle sides and the edges confining gaps.

Stimuli were randomly distributed over a 20° × 20° area around the fixation cross. It was ensured that an equal number of elements appeared in each screen half (left, right, above, and below the fixation cross). Locations of individual stimuli in each trial were drawn such that the Euclidean distance between centers of all displayed stimuli (including the fixation cross) was at least 5°. This value was chosen based on pilot data from one experienced observer, and was intended to ensure that neighboring stimuli were spaced such that participants were unlikely to be able to discriminate targets from distractors with peripheral vision.

### Design

To study the combined influence of prospective search costs and discrimination accuracy on target selection, we designed a combined visual search and perceptual discrimination task. We used a within-subject design with two conditions: the single-target and double-target condition. Data for both conditions was collected in one session, with the order of conditions being fixed (always single-target first). The color of the circle surrounding the rectangles was balanced between participants: for even participant numbers, easy elements were surrounded by a red circle and difficult elements by a blue circle. This pattern was flipped for odd participant numbers. Target orientation was randomized over trials, so, in each trial, a target could be oriented either horizontally or vertically, but their orientation was never the same when both targets were shown in the double-target condition. We manipulated the number of easy/difficult distractors per trial (nine levels, 0–8 easy/difficult distractors per trial) and the discrimination accuracy of targets (two levels, easy and difficult).

Both conditions had a variable number of trials and participants were told to complete as many trials as they could within a timeframe of 6 min and 30 s. To ensure that we collect roughly an equal number of trials for each combination of factor-levels, trials were presented as miniblocks. In the single-target condition, each miniblock was made up of 18 trials (one target per trial (easy or difficult) with 0–8 distractors from the same set as the shown target), presented in a random order. In the double-target condition, each miniblock consisted of nine trials (two targets and 0–8 easy/difficult distractors per trial).

### Procedure

#### Single-target condition

At the start of each trial, a fixation cross appeared at screen center and participants could start trials by pressing the space-bar (Fig. 1B). After a random time interval between 500 ms and 1,000 ms, drawn from a uniform distribution, the stimulus array appeared, while the fixation cross stayed on the screen.

In each trial of the single-target condition, the stimulus array contained one target (easy or difficult) as well as a variable number of distractors (0–8) from the same set as the shown target. Participants were instructed to locate the target and to discriminate the position of a gap, located on one of the two long sides of its rectangular part, by pressing one of the arrow keys on the keyboard. Participants were explicitly told which stimulus set (red or blue) had smaller/larger gaps. Individual trials had no time limit and participants could take as much time as they deemed necessary to locate and discriminate the target. However, each stimulus on the screen (excluding the fixation cross) could only be viewed for up to 500 ms (see *Eye movement and data analysis*), before the currently fixated element was replaced by a mask stimulus with the same ring color as the currently fixated element (participants were instructed about the limited viewing time). If an element was fixated for less than 500 ms, the difference to the remaining time was used as an updated maximum viewing time for the next fixation of the same element. The search display remained visible until a response was provided and participants could respond immediately after stimulus array onset (i.e., without ever fixating any of the elements on the screen). To reduce accidental terminations of trials, the active response keys were confined to the orientation of the target in a trial (e.g., if the target orientation in a trial was vertical, only the left and right arrow keys were active).

After a response was detected, the stimulus array and fixation cross disappeared and visual feedback was shown at screen center. The feedback consisted of the score for the current trial as well as an overall score, which was translated to a monetary payout at the end of the condition (e.g., “0.02 | 0.44”); for each correct discrimination participants received two points (+0.02) and they lost two points (−0.02) for each incorrect response. Additionally, a timer was shown under the score, indicating how much time was left for the task (e.g., 00:06:00). The remaining time was updated after each trial by subtracting the time elapsed between stimulus array onset and response from the remaining time. If participants did not look at the fixation cross at stimulus onset (see *Eye movement and data analysis*), a red error message (“Not fixated!”) was shown instead of the visual feedback and timer. In this case, participants lost two points, irrespective of their actual response, and the remaining time was updated, just as in trials without fixation errors. The feedback stayed on for 1,500 ms, after which it was replaced by a fixation cross and the next trial began. Before the task, participants performed ten demonstration trials.

The purpose of the single-target condition was to familiarize participants with the stimuli and the task. Furthermore, the single-target condition allowed us to quantify a participant’s discrimination performance and search behavior for each target option in isolation.

#### Double-target condition

The double-target condition was identical to the single-target condition, except for the composition of the stimulus array and some aspects of the task. Instead of one target, always two targets were shown per trial (easy and difficult), which were accompanied by 0–8 easy distractors, leading to 1–9 easy elements (*N*_*E*_), and 8–0 difficult distractors, leading to 9–1 difficult elements (*N*_*D*_) (Fig. 1C). Therefore, the stimulus array contained ten elements in each trial, from which two were targets and eight were distractors. However, the exact composition of the elements (*N*_*E*_ > *N*_*D*_, *N*_*E*_ < *N*_*D*_, *N*_*E*_ = *N*_*D*_) varied randomly over trials. Participants were instructed that they could freely choose which target they wanted to search for and discriminate. As in the single-target condition, participants could respond right after stimulus onset, however, in the double-target condition, all response keys were active in each trial (since each trial contained two targets with different orientations). Participants, again, performed ten demonstration trials before the condition.

Although the double-target condition has some notable similarities to a paradigm previously used by Irons and Leber (2016, 2018) (freedom to choose between competing targets that varied in their relative search costs), our paradigm also differed in several critical aspects to the paradigm from Irons and Leber (2016, 2018). First, time to complete trials as well the maximum viewing duration of individual stimuli was limited in our paradigm, whereas both of those factors were unlimited in the paradigm by Irons and Leber (2016, 2018). We introduced those changes to create temporal urgency for participants, and to encourage changes in the preferred target when the search costs of a target (i.e., the required time to find it) became too high. Second, we manipulated the discrimination difficulty of targets, whereas the study by Irons and Leber (2016, 2018) kept discrimination difficulty of target options constant. This addition was a direct consequence of our research question. Third, the distractor ratio in the paradigm by Irons and Leber (2016, 2018) changed in a predictable manner, whereas the relative number of easy and difficult distractors in our paradigm varied unpredictably from trial to trial. We used unpredictable stimulus conditions because we wanted to minimize the impact of statistical learning (i.e., participants learning the pattern in which relative set sizes are presented) and anticipation on our results. Finally, participants in our paradigm received monetary rewards for correct discriminations, whereas the participants from Irons and Leber (2016, 2018) were not rewarded for performance. We introduced this additional factor to motivate participants, and to have a performance measure, which participants could actively influence via more or less sensible target decisions in our paradigm.

The purpose of the double-target condition was to measure if participants take into account the relative discrimination accuracy as well as prospective search costs of target options, when deciding which target to search for and discriminate.

### Eye movement and data analysis

#### Online tracking of element viewing time

In both conditions, participants could view each element in a trial for up to 500 ms before it was replaced by a mask. To determine how long individual elements have been viewed we defined circular areas of interest (AOIs) around the centers of elements on the screen (diameter: 5°, non-overlapping; invisible to participants) and tracked online for how long the measured raw gaze position was located within each AOI. Our algorithm tracked when gaze entered as well as left an AOI and for how long it dwelled within it, but not how the AOI was entered/left. Thus, both instances where gaze actually dwelled within an AOI and where it merely passed through an AOI mid-flight (e.g., during saccades to different elements) were treated as AOI visits and subtracted from the viewing time of the corresponding element.

#### Offline eye-movement analysis

On- and offsets of saccades and blinks were detected using the EyeLink algorithm. The main concern of the offline analysis of eye-movement behavior in our paradigm was to determine which elements participants fixated in a trial while searching for targets. Since both saccades and blinks could shift gaze, and thus change which element participants fixated, both eye-movement types were analyzed together. For offline analysis of viewed elements, we used the same logic as for online tracking of element viewing time (see *Online tracking of element viewing time*). To render our analysis robust against recording errors and random fluctuations in gaze position (e.g., after saccades), we used the average gaze position between offset and onset of consecutive gaze shifts to determine if gaze was located within an AOI between gaze shifts or not. If the average gaze position was located within an AOI, we flagged the corresponding element as viewed and the consecutive gaze shifts as causes for the AOI being entered and left, respectively. If gaze was located within an AOI more than once per trial, each instance was treated as an independent AOI visit. Thus, each visit to an AOI was treated as a separate event, and our analysis does not account for factors such as integration of visual information over multiple fixations of one and the same stimulus.

We excluded gaze shifts with durations shorter than 5 ms, with offsets after stimulus array offset, with onset/offset coordinates outside screen bounds and corrective gaze shifts within AOIs (i.e., small gaze shifts that did not change the currently viewed AOI) from analysis. Additionally, last gaze shifts in trials (i.e., before a response) were excluded if they targeted a location outside of any specified AOI (i.e., the screen background).

We excluded trials from analysis in which participants’ gaze deviated more than 2° from the fixation cross within a timeframe of −20 ms to 80 ms relative to stimulus onset. Additionally, some trials had to be excluded due to technical difficulties during data recording. Applying those criteria left, on average, 96.83% (single-target condition; min.: 89.35%, max.: 100.00%) and 95.73% (double-target condition; min.: 82.91%, max.: 100.00%) valid trials.

#### Calculating planning, inspection, and response time

Planning times were calculated trial-wise as the time between onset of the stimulus array and offset of the first gaze shift after stimulus array onset. Inspection times were also calculated trial-wise by, first, determining which elements were viewed in a trial, second, calculating for each viewed element how long gaze dwelled in the respective AOI around it, and third, averaging the resulting vector of individual dwell times to obtain the average inspection time over all elements in a trial. The dwell time within an AOI was calculated by tracking the time between the gaze entering and leaving the AOI around an element. To account for differences in information uptake when an AOI was left via saccade or blink, dwell times were calculated slightly differently for those cases: When an AOI was left via a saccade, dwell time was calculated as the time between offset of the entering gaze shift (irrespective of if it was a saccade or blink) and offset of the leaving saccade. However, if an AOI was left via a blink, dwell time was calculated as the time between offset of the entering gaze shift (again, irrespective of if it was a saccade or blink) and onset of the leaving blink. If a participant blinked during an AOI visit (i.e., average gaze position was within the same AOI before and after the blink), the duration of the blink was subtracted from the corresponding dwell time to account for interruptions in information uptake during the blink. In the single-target condition, no inspection times were calculated for gaze shifts to targets. In the double-target condition, inspection times for targets were calculated for all instances except for when the last gaze shift in a trial landed on a target.

Finally, response times were calculated trial-wise as the time between offset of the last gaze shift before response (i.e., button-press, indicating the location of the gap) and the time of response. If the last gaze shift landed on a distractor instead of either of the targets, no response time was calculated (single-target condition: 89.19% trials with valid response times, min.: 32.23%, max.: 98.15%; double-target condition: 91.09% trials, min.: 77.32%, max.: 100.00%). If the response was given during a gaze shift that left the AOI around a target or if the response was given after a gaze shift to screen background (i.e., area outside any defined AOI), the corresponding gaze shift was ignored and the cases treated as if the target was fixated until response.

#### Statistical analysis

Two-sided paired-sample *t*-tests as well as two-sided one-sample *t*-tests were used for inference statistics. Normality assumptions were tested using the Lilliefors-test, with exact *p*-values being determined by Monte-Carlo approximation with a maximum Monte-Carlo standard error of 0.001. For calculations of proportion gaze shifts as well as accuracy, planning, inspection and decision times we first calculated the corresponding variable for each set size condition individually, and second averaged over the resulting vector to obtain the final variable estimate for a participant.

### Modeling

Modeling was used to represent the proportion of choices for the easy and the difficult target and the resulting gain as well as the proportion of fixations on the chosen set. We assumed that participants generally acted like an ideal observer, who knows about its individual performance limitations and who can accurately estimate which target in a trial of the double-target condition will yield a higher monetary gain per unit of time. We assumed that unsystematic noise corrupted gain estimates, leading to occasional choices for lower-gain targets. Additionally, we assumed that participants, while searching for targets, had a variable bias to fixate elements from the set of the chosen target, which sometimes resulted in fixations on elements from the set of the non-chosen target.

**Fig. 2:**
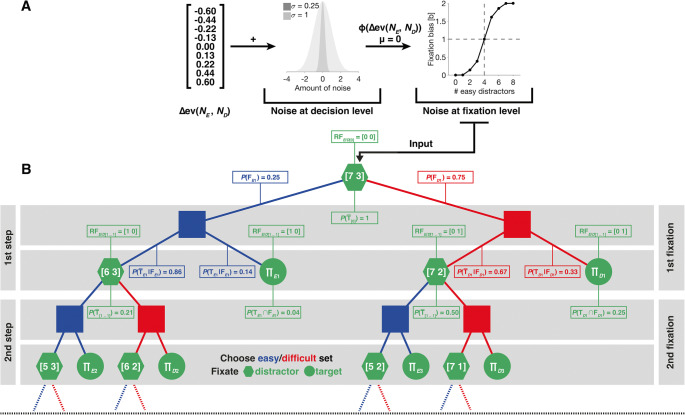
Model schematics. (**A**) Our model received relative expected values of target options under different relative set sizes, as calculated by Equation 3 (see Online Supplementary Material (OSM)), as input. In a first step, gain estimates were corrupted by decision noise, drawn from a normal distribution with standard deviation as a free parameter. This allowed for occasional choices for lower-gain targets. Noisy gain estimates were, in a second step, transformed by a cumulative Gaussian distribution function with its standard deviation as a free parameter; by this, we allowed for occasional fixations on elements of the non-chosen set. The fixation bias parameter, obtained by this transformation, was then used as input to the generative stochastic model, in order to calculate model predictions. (**B**) The generative stochastic model was implemented as a binary decision tree, which was traversed recursively. Here, each recursion step (horizontal gray bars) corresponds to one fixation a participant could make: At each recursion step/fixation, the model chooses to fixate either an element from the easy (blue elements) or difficult set (red elements) and each fixation could either land on a distractor (green polygon) or a target (green circle). After each fixation, one element is removed from the fixated set (numbers inside polygons indicate the remaining set elements after fixation). Recursion terminates when a target was fixated. Predictions for the probability to choose an easy target were obtained by summing over all instances where a target was found (product symbols in green circles). For illustration purposes, the decision tree is only shown partially and terminates prematurely at the black dashed line. For the first recursion step, example values for the results of Equations 7–18 and Equations 23–24 (see OSM), given a search display with seven easy and three difficult distractors and a fixation bias of 1.86 for this particular relative set size, are provided

We modeled this general framework in two steps: First, we used performance data of individual participants and generated, for each set size condition individually, ideal observer predictions about the relative monetary gain per unit of time of available target options. This allowed us to quantify which target participants should search for, in order to maximize their individual gain. In a second step, the ideal observer predictions were used as input to a generative stochastic model, where they were corrupted by noise (Fig. 2A). For this, the generative stochastic model had two free parameters: one free parameter controlled noise that corrupted the gain estimates, provided as input. By this, we allowed our model to occasionally choose lower-gain targets. A second free parameter was used to control the transformation of noisy gain estimates into a set-size-condition-specific fixation bias. During this transformation, additional noise was added that corrupts estimates of participants’ individual fixation biases. By this, we allowed our model to occasionally fixate elements from the set of the non-chosen target during search. Critically, the generative stochastic model only received gain estimates as input and thus ignored factors such as the position of elements on the screen and their spatial distance relative to each other.

The generative stochastic model was implemented as a decision tree, which simulates the series of fixations a participant makes while searching for targets (Fig. 2B). This allowed us to calculate the probabilities of all possible search outcomes (i.e., finding a target with the first, second, … *i*^*th*^ fixation, after having fixated zero, one, two, etc. easy and difficult distractors) under different set size conditions and under varying amounts of noise. The final outputs of the generative stochastic model were two predictions: one for the proportion of cases in which participants chose to discriminate easy targets, and a second prediction of the proportion of fixations on elements of the chosen set. To evaluate model performance, we compared those predictions to the empirical data from the double-target condition. For a more extensive formal description of our generative stochastic model, we would like to refer the reader to the OSM.

## Results

### Gap size and set size influence discrimination performance and search time

Participants in the single-target condition completed, on average, 160 trials within the provided time frame of 6 min and 30 s (min.: 94 trials, max.: 231 trials) and received an average bonus payout of 1.57€ (min.: 0.04€, max.: 3.66€, CI_95%_ [1.08, 2.07]). To get insights into how participants completed trials, we analyzed eye-movement behavior and which elements were fixated while searching for targets. Since the currently fixated element could change as both the consequence of saccades as well as blinks, both types of eye movements were analyzed together and are referred to as gaze shifts from here on now (see *Methods* for details).

**Fig. 3:**
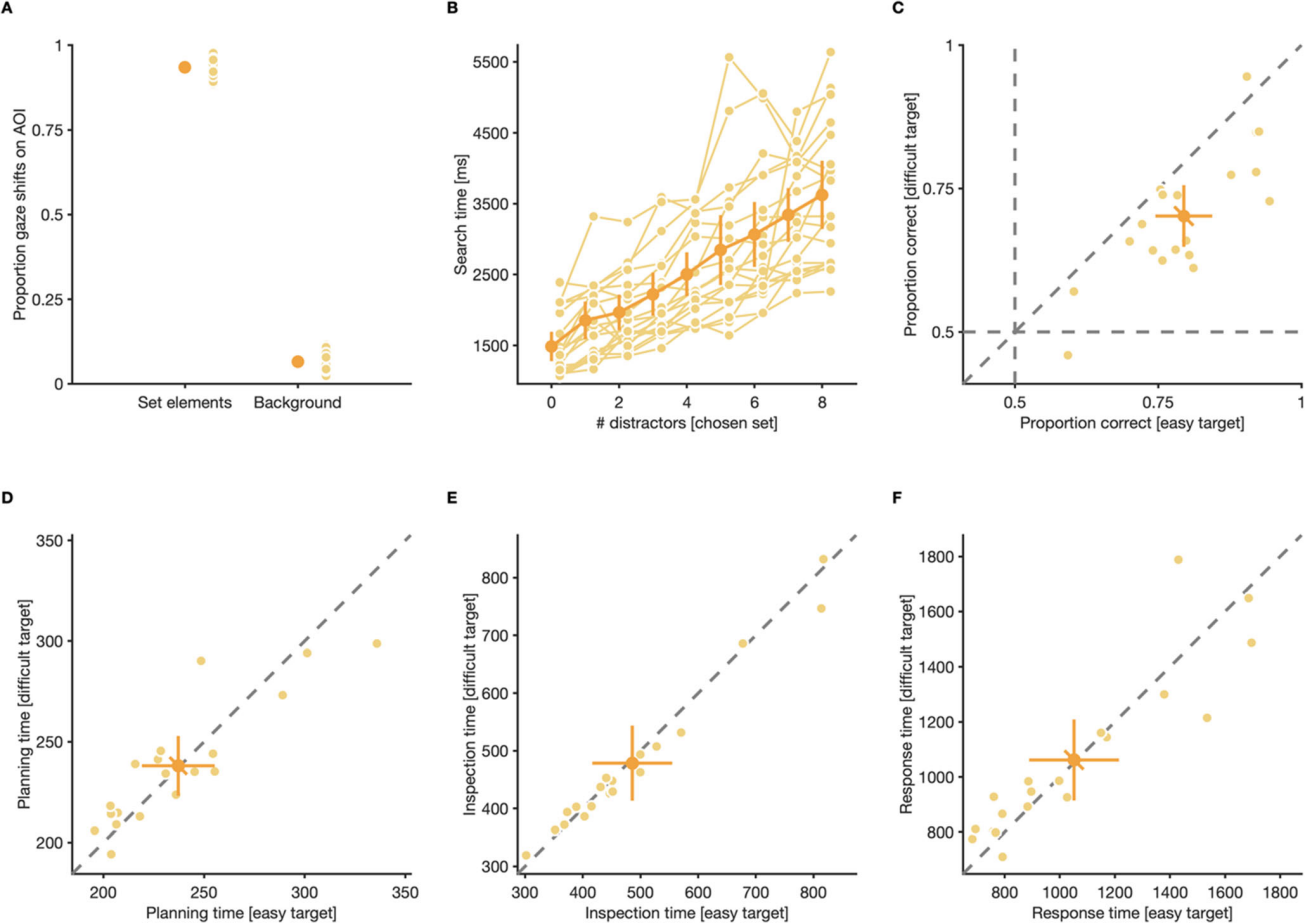
Search behavior and perceptual performance in the single-target condition. (**A**) Proportion gaze shifts that landed within areas of interest (AOIs) around elements from the presented set, and the background. (**B**) Search time (average time between onset of stimuli and response) for different distractor numbers. Search times for easy and difficult targets were analyzed together. (**C**) Discrimination performance, (**D**) planning time (time between stimulus array onset and offset of the first gaze shifts in a trial), (**E**) inspection time (average time between entering and leaving AOIs around elements), and (**F**) response time (time between offset of the last gaze shift in a trial and response) for easy and difficult targets. (**A**–**F**) Small, light dots are data from individual participants, large, dark dots are means across participants. Error bars are 95% confidence intervals. Note the different scales of the y-axes in panels B and D–F

Generally, participants made at least one gaze shift to one of the shown elements in the majority of trials (99.97% of trials with at least one gaze shift to any element), indicating that participants did not tend to systematically skip over trials that were deemed too difficult, either because of too many distractors or because the trial contained a difficult-to-discriminate target. From all gaze shifts, the vast majority targeted AOIs around one of the two element types (targets and distractors) (*M* = 0.93, CI_95%_ = [0.92, 0.95]), while only a minority of gaze shifts landed on the screen area outside any defined AOI (i.e., the screen background: *M* = 0.07, CI_95%_ = [0.05, 0.08]) (Fig. 3A). Thus, the vast majority of the gaze shifts were meaningful and intended to sample visual information from search displays.

Since stimuli were designed such that targets could only be discriminated from distractors with foveal vision, we expected that visual search in our paradigms progresses serially, and that the time participants spent searching for targets (search time), thus, should increase linearly as a function of the number of distractors shown (for examples on how search times increase logarithmically during parallel search, see Buetti et al., 2016; Ng et al., 2018). To quantify search time, we tracked how much time passed between stimulus onset and response in individual trials and calculated the average of the resulting vector for each participant and set size condition. Analyzing the resulting search time, without separating for which target-difficulty was shown, revealed that participants, on average, spent more time searching for targets the more distractors were shown (Fig. 3B), with each additional distractor on the screen increasing search time for targets by *M* = 264 ms, CI_95%_ = [225 ms, 302 ms], *t*(18) = 14.49, *p* < 0.001. This value corresponds to roughly half of the time participants, on average, spent fixating elements of either set (inspection time without separating for target difficulty: 482 ms, CI_95%_ = [415 ms, 550 ms]) before finding a target. This result serves as a manipulation check that participants, as intended by our stimulus design, had to fixate elements individually while searching for targets among distractors: Since we assume that an ideal observer has to inspect half the distractors in a trial before finding a target, each additional distractor on the screen should increase the required number of inspected distractors by 0.50 elements, and thus increase search time by half of the inspection time per element. Furthermore, this also serves as evidence that targeting locations in-between elements, in order to assess the identity of multiple elements at once (Eckstein et al., 2015; Najemnik & Geisler, 2005), was not functional in our paradigm.

After finding a target, most participants had, in accordance with our intended manipulation of discrimination difficulty, a lower perceptual performance when discriminating difficult (proportion correct *M* = 0.70, CI_95%_ [0.65, 0.76]) compared to easy targets (*M* = 0.79, CI_95%_ [0.75, 0.84]), *t*(18) = 5.84, *p* = < 0.001 (Fig. 3C). Other than that, participants did not behave significantly different when searching and discriminating different targets: they spent a comparable amount of time planning their search at the start of trials, irrespective of which target they eventually had to discriminate (planning time; easy target: *M* = 237 ms, CI_95%_ [219 ms, 255 ms]; difficult target: *M* = 238 ms, CI_95%_ [223 ms, 253 ms]) (Fig. 3D), *t*(18) = −0.22, *p* = 0.831, they spent a comparable amount of time inspecting elements in-between successive gaze shifts (inspection time; easy target: *M* = 486 ms, CI_95%_ [417 ms, 555 ms]; difficult target: *M* = 479 ms, CI_95%_ [413 ms, 544 ms]) (Fig. 3E), *t*(18) = 1.35, *p* = 0.195, and it took them comparably long to discriminate gap positions on both target types (response time; easy target: *M* = 1,052 ms, CI_95%_ [889 ms, 1,215 ms]; difficult target: *M* = 1,062 ms, CI_95%_ [914 ms, 1,209 ms]) (Fig. 3F), *t*(18) = −0.30, *p* = 0.768.

Taken together, results from the single-target condition demonstrate that participants, as intended by our stimulus design, had to fixate elements individually while searching for targets among distractors. Furthermore, results from this condition demonstrate that both our manipulations were successful: Participants spent more time searching for targets the more distractors of the same set were shown and they had a lower perceptual performance when discriminating targets from difficult, compared to easy sets. Critically, although different gap sizes influenced perceptual performance, they did not influence search behavior, with participants spending a comparable amount of time planning their search, inspecting elements in-between gaze shifts, and making perceptual decisions for both set types.

### Discrimination accuracy and search costs are traded off when choosing targets

Whereas we showed either the easy or difficult target in the single-target condition, accompanied by a varying number of distractors from the same set, both targets and a mix of easy and difficult distractors were presented in the double-target condition. Thus, here, participants not only had to find and discriminate targets, but they also had to make a series of decisions in each trial: (a) which target they want to search for, and (b) which elements they want to fixate during search for the chosen target. Under these more challenging conditions, participants, on average, completed 180 trials within the provided time frame (min.:: 103 trials, max.: 245 trials) and received an average bonus payout of 2.12€ (min.: −0.02€, max.: 4.02€, CI_95%_ [1.57, 2.67]), which was significantly higher than the bonus payout in the single-target condition, *t*(18) = −4.73, *p* < 0.001 (see OSM Fig. S1 for search behavior and perceptual performance in the double-target condition). As in the single-target condition, at least one shown element was fixated in the majority of trials (99.98% of trials with at least one gaze shift to any element), indicating that participants, again, did not utilize a systematic strategy to skip over certain trials. Participants in the double-target condition thus completed more trials and accumulated a higher bonus payout compared to the single-target condition. This alone implies that discrimination accuracy and search costs of target options in the double-target condition must have been taken into account to optimize which target is discriminated, otherwise we would have expected a similar performance as in the single-target condition.

**Fig. 4:**
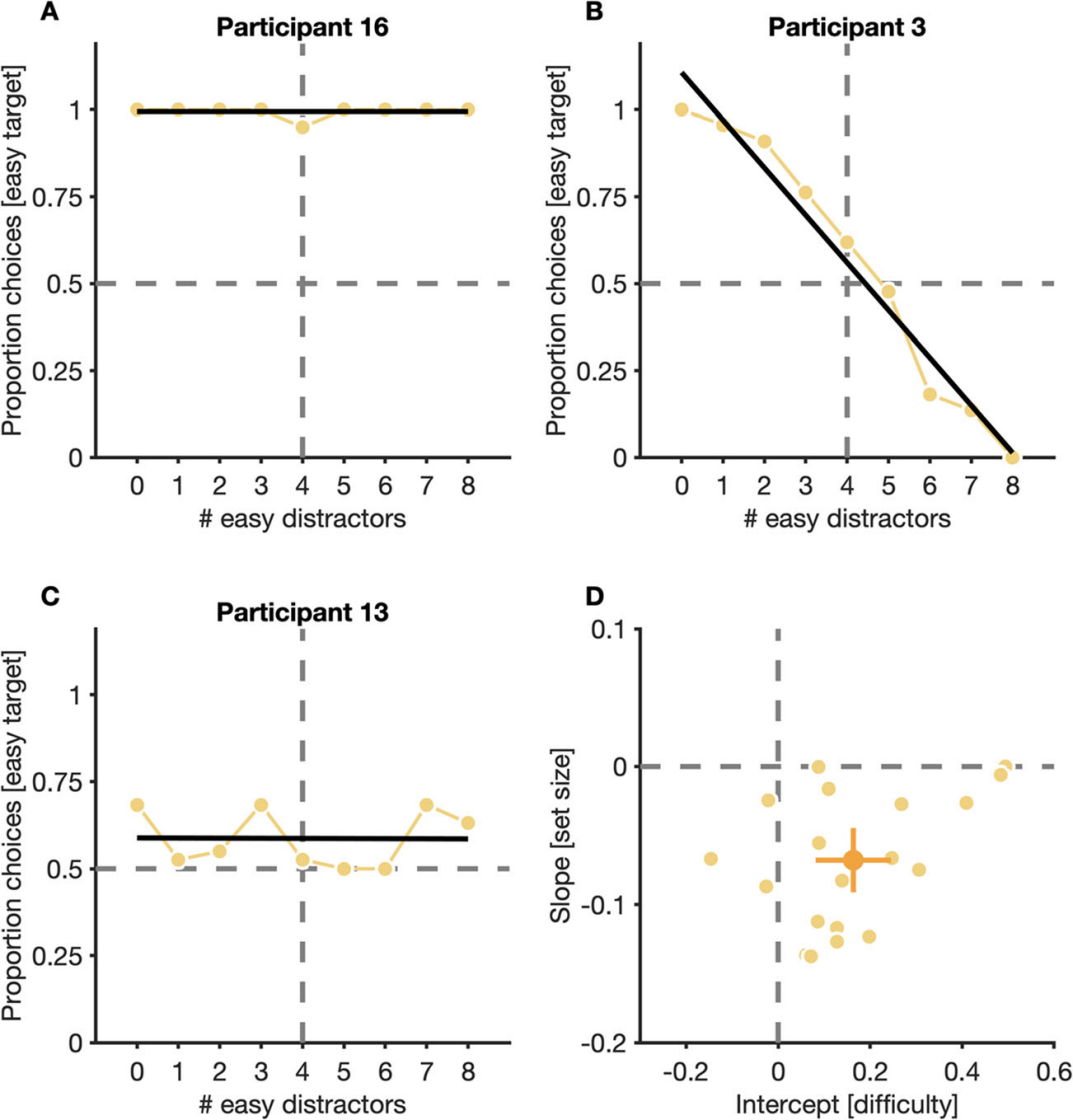
Choices for targets in the double-target condition. (**A**–**C**) Proportion of trials in which three representative participants chose to discriminate easy targets, separately for different relative numbers of easy and difficult distractors. A minority of participants always chose to discriminate easy targets (**A**), irrespective of the relative number of easy distractors in trials, most participants adapted their behavior to changes in the relative number of easy and difficult distractors (**B**), and some participants showed no discernable pattern in their choice behavior (**C**). Small, light dots are proportions for individual participants, black lines are fits of linear regressions. (**D**) Slopes and intercepts of linear regressions, fitted to the choice curves of individual participants. Small, light dots are data from individual participants, the larger, dark dot is the mean across participants. Error bars are 95% confidence intervals

To get a more fine-grained insight into how search costs and discrimination accuracy affected participants’ choice behavior in the double-target condition, we analyzed which target they chose to discriminate under different set size conditions. When given the freedom to choose which target to discriminate, participants, similar to previous studies (Irons & Leber, 2016), showed large interindividual differences in their choice behavior: A minority of participants showed a strong preference for easy targets (2 out of 19 participants) and this preference persisted irrespective of the relative number of easy and difficult distractors in trials (Fig. 4A; see also participant 20 in OSM Fig. S2). However, other participants showed, to varying degrees, changes in their preferred target: They preferred easy targets when only a few easy distractors were shown, and they gradually switched their preference to difficult targets the more distractors from the easy set were shown (Fig. 4B). Only some of the participants (3 out of 19 participants) showed no discernable pattern in their choice behavior, with choice-curves fluctuating around chance across all relative set sizes (Fig. 4C; see also participants 10 and 19 in OSM Fig. S2).

To choose targets participants could base their decisions on multiple sources of information: First, they could base their decision entirely on target difficulty and, by this, maximize the probability of correct target discriminations after search. Second, participants could choose targets purely based on the relative number of easy and difficult distractors in trials, that is, the temporal costs that come with searching for a target in the corresponding set. Searching for targets in sets with less elements would allow participants to minimize search costs per trial and, by this, to maximize the number of completed (although not necessarily correctly discriminated) trials. Finally, participants could take into account both search costs as well as discrimination accuracy, and select targets that have the higher ratio between prospective search costs and probability of correct discrimination, that is, targets with higher monetary gains per unit of time.

To quantify which factors contributed to the choice behavior of participants, we fitted linear regressions to the choice curves of individuals and analyzed slopes and intercepts of the resulting fits. The regression of easy target choices on number of easy distractors was normalized such that an intercept of zero corresponds to a perfectly balanced choice behavior (i.e., 50% easy choices at four easy and four difficult distractors). Therefore, chance performance (0.50) was subtracted from empirical proportion choices for easy targets, and four was subtracted from the actual number of easy distractors before fitting. We found that both average intercepts (*M* = 0.16, CI_95%_ [0.08, 0.25]), *t*(18) = 4.20, *p* = 0.001, and average slopes (*M* = −0.07, CI_95%_ [−0.09, −0.04]), *t*(18) = −6.09, *p* = < 0.001, differed significantly from zero (Fig. 4D). More specifically, intercepts of a notable subset of participants showed a positive shift away from zero, implying that those participants tended to prefer easy over difficult targets, whereas slopes of a notable subset of participants showed a negative trend, suggesting that those participants preferred targets with lower over targets with higher search costs (see OSM Fig. S2 for regression fits of all participants in the double-target condition).

In summary, results from the double-target condition show that a majority of participants, when choosing targets, not only considered the relative discrimination accuracy or the relative search costs of target options in trials. Instead, a notable subset of our participants took into account both of these factors and dynamically adapted their preference for targets to unpredictable changes in the relative search costs of easy and difficult targets across trials.

### Fixations during search in the double-target condition favor elements from both stimulus sets

Besides choosing targets, participants also had to decide which elements to fixate while searching for the chosen target in the double-target condition. To check which elements were fixated during visual search, and if participants, in accordance with ideal observer predictions, restricted fixations to elements from the set of the chosen target, we analyzed which elements were fixated over the course of trials in the double-target condition. Since the number of executed gaze shifts per trial could vary drastically, depending on the individual participant, the chosen target, and the relative set size, we restricted our analysis to the first two gaze shifts in trials.

**Fig. 5:**
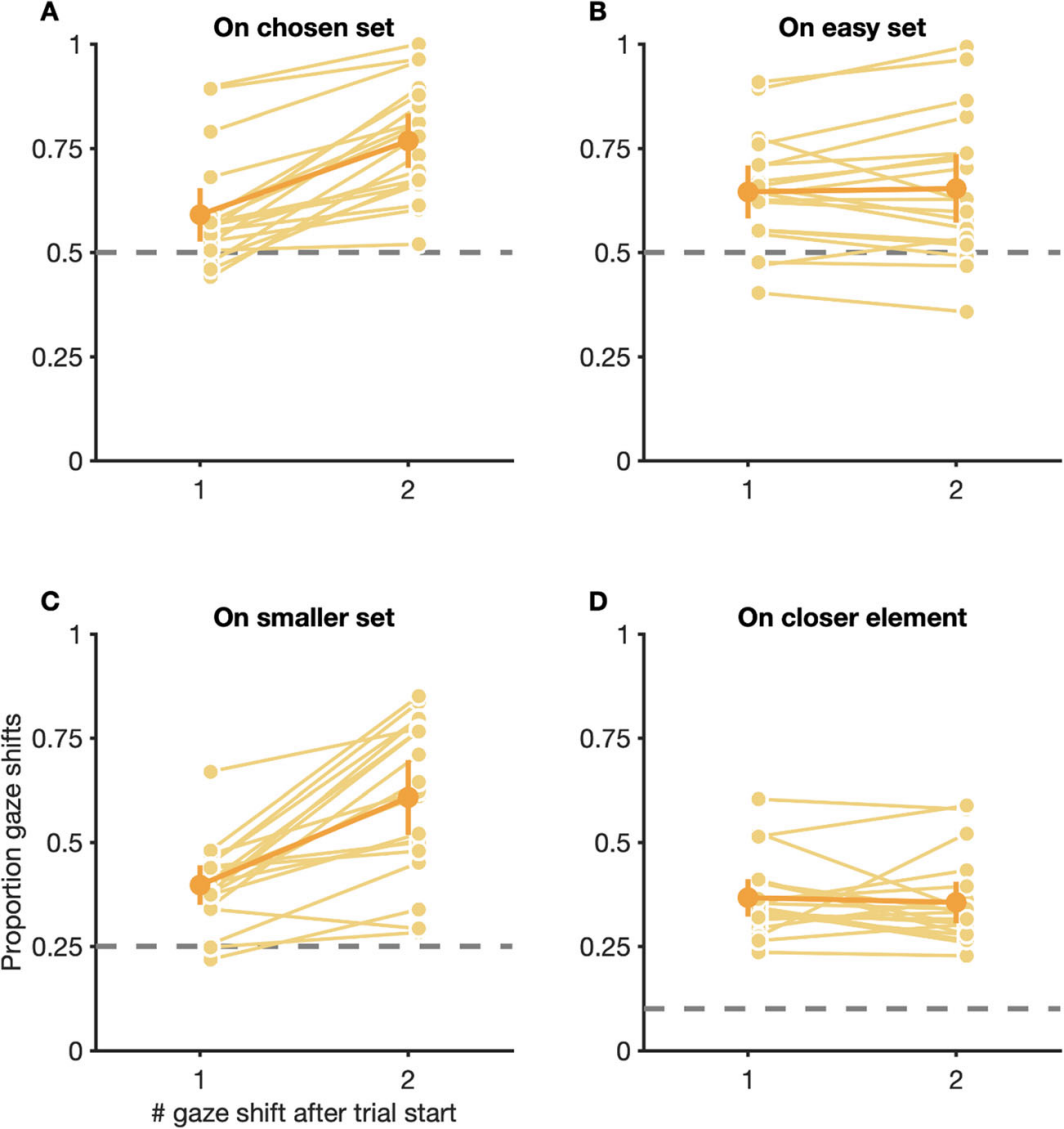
Choices for fixation locations of the first two gaze shifts in trials in the double-target condition. Proportion of gaze shifts to elements belonging to the set of the eventually chosen target (**A**), elements belonging to the set of the easy target (**B**), elements belonging to the set with less distractors (**C**), elements closest to the current fixation location (**D**). (**C**) Conditions with equal numbers of easy and difficult distractors were excluded when analyzing proportion gaze shifts to the smaller set. (**A**–**D**) Gaze shifts to the background were excluded when calculating proportions. Small, light dots are data from individual participants, large, dark dots are means across participants. Error bars are 95% confidence intervals. Dashed gray lines are chance levels

Our analysis revealed that, for first gaze shifts in trials, fixations on average showed a slight preference for elements belonging to the set of the eventually chosen target (proportion first gaze shifts on chosen set: *M* = 0.59, CI_95%_ [0.53, 0.65]; chance level: 0.50) and this preference became significantly more pronounced for second gaze shifts in trials (*M* = 0.77, CI_95%_ [0.70, 0.83]), *t*(18) = −7.76, *p* < 0.001 (Fig. 5A). Similarly, first gaze shifts in trials on average also showed slight biases towards elements belonging to the smaller set (proportion first gaze shifts on smaller set: *M* = 0.40, CI_95%_ [0.35, 0.45]; chance level: 0.25), which, once again, became more pronounced later in trials (*M* = 0.61, CI_95%_ [0.52, 0.70]), *t*(18) = −6.28, *p* < 0.001 (Fig. 5C). Additionally, constant (and interindividually variable) biases towards elements from the easy set (proportion first gaze shifts on easy set: *M* = 0.65, CI_95%_ [0.58, 0.71]; second gaze shifts: *M* = 0.65, CI_95%_ [0.57, 0.74]; chance level: 0.50), *t*(18) = −0.48, *p* = 0.636 (Fig. 5B), and elements closest to the current fixation location (proportion first gaze shifts to closest element: *M* = 0.37, CI_95%_ [0.32, 0.41]; second gaze shifts: *M* = 0.36, CI_95%_ [0.31, 0.41]; chance level: 0.10), *t*(18) = 0.52, *p* = 0.612) (Fig. 5D), were observed throughout trials.

To summarize, although participants, on average, showed a general bias to preferentially fixate elements from the set of the ultimately chosen target, a substantial proportion of gaze shifts was directed to elements from the set of the non-chosen target. Although this preference was most pronounced for first gaze shifts in trials, it also persisted, to a smaller degree, even later in trials.

### Noise at the decision and fixation level limits gain in the double-target condition

**Fig. 6:**
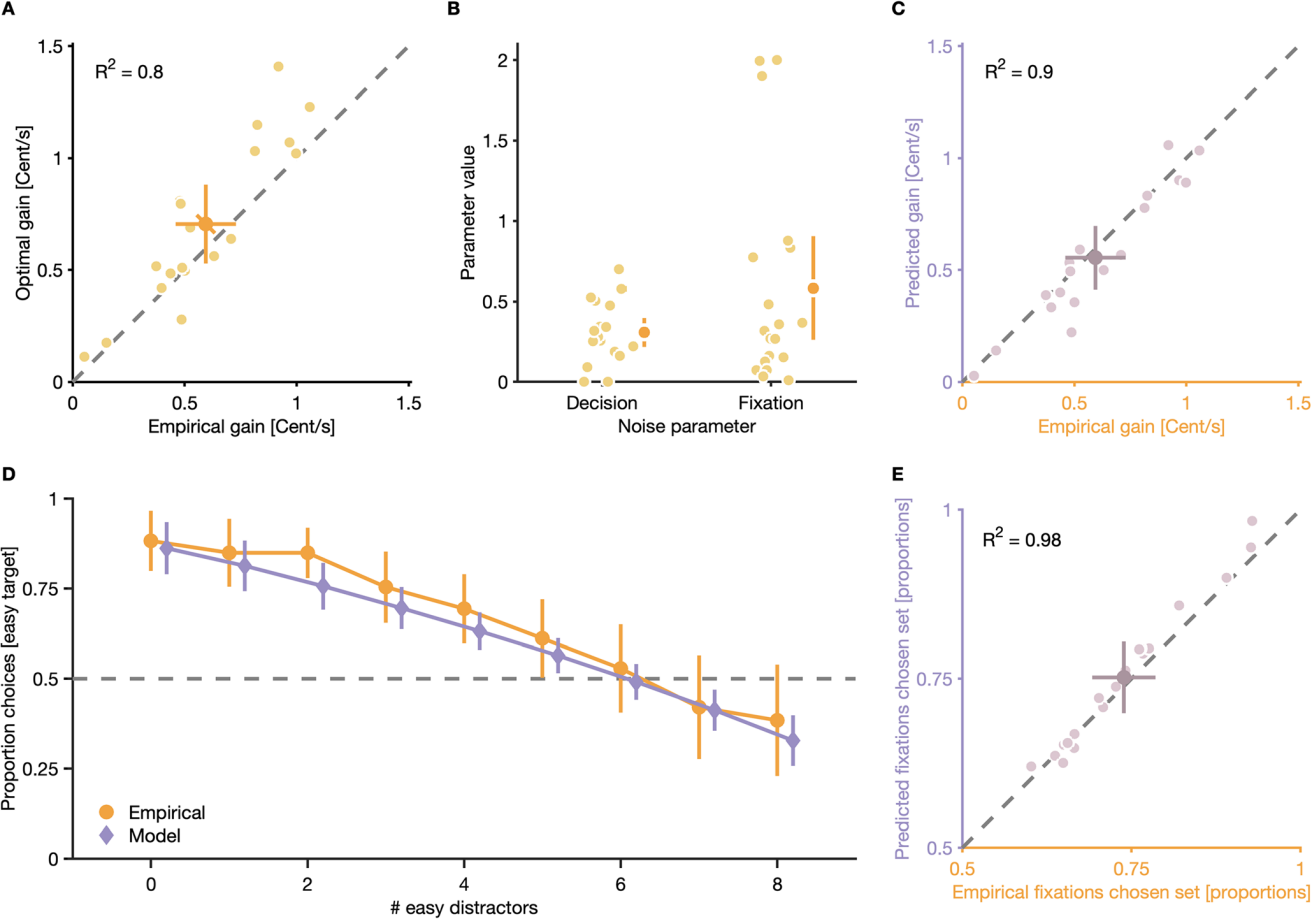
Results of model fit. (**A**) Comparison between empirical gain and theoretically possible gain if participants always discriminated higher-gain targets and restricted fixations during visual search to elements from the set of the chosen target. Empirical gain was calculated as the ratio between accumulated reward (bonus payout in the double-target condition) and the time participants had to complete the task (the time provided to complete as many trials as participants could). (**B**) Distributions of free model parameters that represent noise at the decision and fixation level. (**C**) Comparison between empirical gain and predicted gain. (**D**) Average empirical proportion choices for easy targets and predicted proportion choices for easy targets. (**E**) Empirical proportion fixations to elements of the chosen set and predicted proportion fixations to elements of the chosen set. (**A**–**E**) Small, light dots are data from individual participants, large, dark dots are means across participants. Error bars are 95% confidence intervals. Note the truncated y-axis in panel E

The optimal solution (i.e., the one that maximizes the individual monetary gain per unit of time) to the task in the double-target condition would be to determine the higher-gain target before making the first gaze shift, and to exclusively inspect distractors from the set of the chosen target until it is found. Empirical results from the double-target condition, however, suggest that participants only partially deployed this optimal strategy: Although the majority of participants, on average, chose higher-gain targets, lower-gain targets were also chosen occasionally (OSM Fig. S2). Furthermore, although participants, on average, preferentially fixated elements from the set of the chosen target, while searching for it, a substantial proportion of gaze shifts also landed on elements from the set of the non-chosen target (Fig. 5). To quantify the impact of such deviations from the optimal strategy on participants’ performance in the double-target condition, we compared their average empirical monetary gain per unit of time in the double-target condition to a theoretical maximum gain they could have achieved if they always deployed the optimal strategy. In line with the observed deviations, participants, on average, achieved a smaller monetary gain per unit of time (*M* = 0.59 Cent/s, CI_95%_ [0.46 Cent/s, 0.73 Cent/s]), compared to what was theoretically possible, given their individual properties of visual search and perception (*M* = 0.71 Cent/s, CI_95%_ [0.53 Cent/s, 0.88 Cent/s]), *r* = 0.90, CI_95%_ [0.75, 0.96]), *p* < 0.001 (Fig. 6A).

To quantify the impact of noise at the decision level (i.e., occasional choices for lower-gain targets) and noise at the fixation level (i.e., occasional fixations on elements from the set of the non-chosen target) on participants performance in the double-target condition, we built a generative stochastic model and fitted it to participant’s data from the double-target condition. Our model relaxes both the assumption that participants can always perfectly estimate which target will yield higher gain, and that participants will restrict fixations during search to elements from the set of the chosen target. Instead, one free parameter introduces decision noise that corrupts relative gain estimates of target options in trials, and thus allows for occasional choices of lower-gain targets. Furthermore, the model treats choices for fixation locations during visual search as a stochastic process, where participants have a variable bias to fixate elements from the set of the chosen target, which allows for some proportion of “random” fixations to elements of the non-chosen set (see *Methods* for details).

Generally, both noise at the decision (*M* = 0.31, CI_95%_ [0.22, 0.40]) and the fixation level (*M* = 0.58, CI_95%_ [0.26, 0.90]) contributed to participant’s fixation locations during visual search and choices for targets (Fig. 6B). Overall, our model not only succeeded in predicting participant’s performance in the double-target condition (empirical gain: *M* = 0.59 Cent/s, CI_95%_ [0.46 Cent/s, 0.73 Cent/s]); predicted gain: *M* = 0.55 Cent/s, CI_95%_ [0.41 Cent/s, 0.70 Cent/s]) (Fig. 6C), *r* = 0.95, CI_95%_ [0.87, 0.98]), *p* < 0.001, but it also captured their average choice behavior for targets (Fig. 6D, see OSM Fig. S2 for individual fits) and eye-movement behavior during visual search (empirical proportion of fixations on elements from the chosen set: *M* = 0.74, CI_95%_ [0.69, 0.79]; predicted: *M* = 0.75, CI_95%_ [0.70, 0.80]) (Fig. 6C; see OSM Fig. S3 for comparisons between empirical and predicted proportion gaze shifts on elements of the chosen set for different set size conditions), *r* = 0.99, CI_95%_ [0.98, 1.00]), *p* < 0.001.

To summarize, modeling results demonstrate that both noise at the decision level, modeled as decision noise that corrupted individual estimates of relative target gain, and noise at the level of fixations during visual search, modeled as a variable fixation bias that allowed for some proportion of random fixations to elements of the non-chosen set, contributed to participants showing a lower performance in the double-target condition than theoretically possible. Thus, although the majority of participants could successfully trade off search costs and discrimination accuracy to discriminate gain-maximizing targets, their final performance in the double-target condition was constrained by noise in decisions and fixations.

## Discussion

Previous studies on target selection during visual search found that humans can dynamically trade off saliency and reward to select targets that maximize expected value (Navalpakkam et al., 2010). Here we investigated if a similar principle applies to more complex scenarios: Participants in our paradigm had to choose between two equally salient targets, which differed in their respective discrimination accuracy and the associated search costs. Since time to complete trials was limited, participants, in each trial, had to weigh the probability of correctly discriminating targets against the required time to find them amidst distractors, in order to search for targets that maximize individual monetary gains per unit of time.

Similar to previous findings (Navalpakkam et al., 2010), we report that participants were, on average, able to dynamically trade off discrimination accuracy and search costs of target-options to search for targets that maximized expected value (Fig. 4). However, despite basing their choice for which target to search for on the relative monetary gains of available target options, participants accumulated slightly less reward than was theoretically possible (Fig. 6A). Our analysis revealed that this was due to deviations from the optimal choice in decision making (Fig. 4) and occasional fixations on elements from the non-chosen set during search (Fig. 5). Using a generative stochastic model with two free parameters, which successfully captured the observed trade off in discrimination accuracy and search costs, we found that noise corrupting subjective gain estimates in decision making and noise in the selection of fixation locations during search were necessary to explain participants’ choice and eye-movement behavior during search (Fig. 6B–E) (a similar approach was recently used to explain target selection during visual foraging (Clarke et al., 2022a) and visual search (Clarke et al., 2016)). Thus, occasional choices for low-gain targets and a failure to perfectly restrict fixations during search to elements from the set of the chosen target diminished participants’ individual monetary gain per unit of time in the double-target condition and caused them to fall short of the theoretically possible gain.

Our findings share some similarities with results from a series of recent studies (Irons & Leber, 2016, 2018) that investigated selection of visual search targets in dynamic environments with multiple target options. Similar to us, Irons and Leber gave participants the freedom to choose between two differently-colored visual search targets, while varying how many of the shown distractors shared a color with one or the other target. The authors reported that some of their participants spontaneously tended to choose targets that came with less distractors, that is, that minimized search time. However, most participants of Irons and Leber (2016, 2018) were sluggish in updating their preferred target to variations in the relative number of distractors and some participants had strong biases to preferentially search for one particular target, which the authors interpret as tendencies to minimize effort instead of search time (Irons & Leber, 2016, 2018; for a review, see Irons & Leber, 2020).

We observed similar variations in choice behavior in our paradigm, with some participants showing strong preferences for easy targets, whereas other participants dynamically adapted their target preference to unpredictable changes in the relative search costs of available target options (Fig. 4, OSM Fig. S2). Whereas Irons and Leber (2016) interpreted those variations as failures of participants to take into account relevant properties of stimulus displays, we would refrain from doing so. Instead, our modelling shows that these individual differences are the consequences of individual performance differences in the discrimination task and speed differences in the search task. For instance, some participants might have experienced the difficult target as too difficult, leading them to adopt a strong preference for the easy target, which they were reluctant to abandon because choosing the easy target still maximized participants' individual gain, given their inability to discriminate the difficult target.

However, our results also show some dissimilarities: Whereas Irons and Leber (2016) reported that their participants chose targets before trial start, and mainly searched within the set of the chosen target from the first fixation onwards, our participants showed a tendency to inspect elements from both stimulus sets, which was most pronounced for the first gaze shift and became weaker for subsequent gaze shifts (Fig. 5). Even late in trials, a substantial proportion of gaze shifts continued to land on elements from the set of the non-chosen target. One reason for this difference might be that the search environments in the study of Irons and Leber (2016) followed a predictable pattern that allowed participants to anticipate which target will be quicker to find in the following trial, well before stimulus onset (see also Hansen et al., 2019). Search environments in our paradigm, however, changed unpredictably between trials and participants only learned about the composition of a search environment after stimulus onset. Thus, in order to optimally react to a given environment, participants in our paradigm would have needed to delay initial gaze shifts in trials until a target was chosen based on peripheral vision and search for the chosen target was planned.

Indeed, target selection for saccades improves when eye movements are delayed (Ghahghaei & Verghese, 2015; Schütz et al., 2012), however, delaying eye movements also requires active inhibition of the initial reaction to suddenly appearing stimuli (Wolf & Lappe, 2020), which might constitute an effortful process. A failure to successfully inhibit gaze shifts might cause premature gaze shifts to elements from suddenly appearing stimulus arrays, executed before a target was chosen and directed to whichever stimulus is closest to fixation (Irons & Leber, 2016), to whatever is the most salient stimulus (Vanunu et al., 2021) or to random distractors, in an attempt to search for all available target options simultaneously (Kristjánsson et al., 2014). Thus, participants in our paradigm did not only have to trade off search costs and discrimination difficulty of target options, but they also had to balance the benefits of cognitive control to restrict fixations to elements from the set of the chosen target against the costly mental effort that is required to maintain such control (see also Petitet et al., 2021; for a review, see Shenhav et al., 2017). Delaying gaze shifts and increasing the time to inspect search environments before starting search (Hansen et al., 2019; Wolf & Lappe, 2020) might improve selection of fixation locations for first as well as subsequent gaze shifts (Caspi et al., 2004) and reduce noise in target selection.

Although selection of fixation locations generally improved later in trials, roughly a quarter of gaze shifts continued to land on elements from the non-chosen set even late in trials. Previous studies reported that humans are generally capable of choosing fixation targets based on their prospective task utility (Stewart et al., 2022) and that eye-movement targets for both individual saccades (Najemnik & Geisler, 2005, 2008) as well as saccade sequences (Hoppe & Rothkopf, 2019) can be chosen such that they maximize information gain after eye movements. However, other studies report contrary findings, with participants failing to optimize information gain after eye movements in a variety of different paradigms (Araujo et al., 2001; Clarke & Hunt, 2016; Morvan & Maloney, 2012; Nowakowska et al., 2017, 2021). Similarly, although short saccade sequences can be planned so that the eye-movement sequence maximizes the final information gain (De Vries et al., 2014; Hoppe & Rothkopf, 2019), longer movement sequences in search tasks are planned for hand movements, but not for saccades (Diamond et al., 2017).

Our data are in line with previous findings reporting features of suboptimality in oculomotor behavior: Instead of exclusively fixating elements that maximize information about the target’s location (i.e., elements from the same set as the chosen target), selection of fixation locations for early and late gaze shifts in our paradigm was also influenced by factors such as proximity of elements to the current fixation location, implying that participants acted shortsightedly when searching for targets (Fig. 5D). Especially strongly unbalanced search displays, where a singular higher-gain target was shown surrounded by multiple elements from the set of the lower-gain target, might thus have promoted selection of fixation locations that corresponded to closer, but lower-gain elements over locations corresponding to the more distant but higher-gain target (Araujo et al., 2001). Furthermore, occasional fixations on elements of the non-chosen set might also be a consequence of a maladaptive exploration/exploitation trade off (Hills et al., 2010). Instead of exploiting the expected value of available target options, search displays might have been explored overly extensively, in order to refresh knowledge about the relative target accuracy or to reduce uncertainty about the search environment, although costing participants valuable time in which more reward could have been accumulated. Finally, fixations on elements from the set of the non-chosen target might also be indicative of a decision process which gradually unfolds over the course of a trial. Although previous research found evidence that participants plan saccade sequences instead of planning each saccade individually (De Vries et al., 2014; Hoppe & Rothkopf, 2019), and that perceptual decision are made before a motor response that communicates the decision (Lisi et al., 2022), fixations on elements from the non-chosen set might correspond to changes of mind about which target to search and discriminate (Resulaj et al., 2009). The fixation noise in our model is unspecific and does neither favor nor exclude the possibility that changes of mind might be the driving force behind fixation on elements from the non-chosen set. However, due to the fact that the noise parameters in our model are unspecific, they might capture changes of mind, and other behavioral heuristics (e.g., preferences for saccades of a given amplitude), implicitly. Future research needs to determine the impact of those factors on performance in our paradigm.

To conclude, we found that a notable subset of our participants could dynamically trade off search costs and discrimination accuracy to search for targets that optimize individual monetary gains per unit of time. This ability might have yielded an evolutional advantage in the past (deciding from which source to forage in the presence of nearby predators) and can still be helpful to adapt behavior to the demands of the modern world (grocery shopping close to closing time). However, it is ultimately limited by noise that corrupts the decision between competing objects of interest, and noise that corrupts what information to sample from the world, while searching for an object of interest.

## Supplementary Information

ESM 1 (DOCX 1.47 mb)

## Data Availability

Data is available from the following doi: 10.5281/zenodo.6573835. The experiment was not preregistered.

## Code availability

MATLAB code to generate ideal observer predictions and to fit the generative stochastic model is available from the following DOI: 10.5281/zenodo.6573835. MATLAB code to run the experiment and MATLAB code of the remaining analysis pipeline is available upon reasonable request.

## References

[CR1] Ackermann JF, Landy MS (2013). Choice of saccade endpoint under risk. Journal of Vision.

[CR2] Araujo C, Kowler E, Pavel M (2001). Eye movements during visual search: The costs of choosing the optimal path. Vision Research.

[CR3] Becker SI (2011). Determinants of dwell time in visual search: Similarity or perceptual difficulty?. PLoS ONE.

[CR4] Bergmann N, Tünnermann J, Schubö A (2020). Which search are you on? Adapting to color while searching for shape. Attention, Perception, and Psychophysics.

[CR5] Brainard DH (1997). The Psychophysics Toolbox. Spatial Vision.

[CR6] Buetti S, Cronin DA, Madison AM, Wang Z, Lleras A (2016). Towards a better understanding of parallel visual processing in human vision: Evidence for exhaustive analysis of visual information. Journal of Experimental Psychology: General.

[CR7] Cain, M. S., Vul, E., Clark, K., & Mitroff, S. R. (2012). A Bayesian optimal foraging model of human visual search. *Psychological Science, 23*(9), 1047–1054. 10.1177/0956797612440460 10.1177/0956797612440460 22868494

[CR8] Caspi A, Beutter BR, Eckstein MP (2004). The time course of visual information accrual guiding eye movement decisions. Proceedings of the National Academy of Sciences of the United States of America.

[CR9] Clarke ADF, Green P, Chantler MJ, Hunt AR (2016). Human search for a target on a textured background is consistent with a stochastic model. Journal of Vision.

[CR10] Clarke ADF, Hunt AR (2016). Failure of intuition when choosing whether to invest in a single goal or split resources between two goals. Psychological Science.

[CR11] Clarke ADF, Hunt AR, Hughes AE (2022). Foraging as sampling without replacement: A Bayesian statistical model for estimating biases in target selection. PLoS Computational Biology.

[CR12] Clarke ADF, Irons JL, James W, Leber AB, Hunt AR (2020). Stable individual differences in strategies within, but not between, visual search tasks. Quarterly Journal of Experimental Psychology.

[CR13] Clarke, A. D. F., Nowakowska, A., & Hunt, A. R. (2022b). Visual search habits and the spatial structure of scenes. *Attention, Perception, & Psychophysics, 84*(6), 1874–1885. 10.3758/s13414-022-02506-2 PMC9338010 35819714

[CR14] Cornelissen, F. W., Peters, E. M., & Palmer, J. (2002). The Eyelink toolbox: Eye tracking with MATLAB and the Psychophysics Toolbox. *Behavior Research Methods, Instruments, & Computers, 34*(4), 613–617. 10.3758/BF03195489 10.3758/bf03195489 12564564

[CR15] De Vries JP, Hooge ITC, Verstraten FAJ (2014). Saccades toward the target are planned as sequences rather than as single steps. Psychological Science.

[CR16] Diamond JS, Wolpert DM, Flanagan JR (2017). Rapid target foraging with reach or gaze: The hand looks further ahead than the eye. PLoS Computational Biology.

[CR17] Eckstein MP (2011). Visual search: A retrospective. Journal of Vision.

[CR18] Eckstein MP, Schoonveld W, Zhang S, Mack SC, Akbas E (2015). Optimal and human eye movements to clustered low value cues to increase decision rewards during search. Vision Research.

[CR19] Egeth HE, Virzi RA, Garbart H (1984). Searching for conjunctively defined targets. Journal of Experimental Psychology: Human Perception and Performance.

[CR20] Ehinger KA, Wolfe JM (2016). When is it time to move to the next map? Optimal foraging in guided visual search. Attention, Perception, and Psychophysics.

[CR21] Foley NC, Kelly SP, Mhatre H, Lopes M, Gottlieb J (2017). Parietal neurons encode expected gains in instrumental information. Proceedings of the National Academy of Sciences of the United States of America.

[CR22] Ghahghaei S, Verghese P (2015). Efficient saccade planning requires time and clear choices. Vision Research.

[CR23] Gottlieb J (2012). Attention, Learning, and the Value of Information. Neuron.

[CR24] Gottlieb J (2018). Understanding active sampling strategies: Empirical approaches and implications for attention and decision research. Cortex.

[CR25] Gottlieb J, Hayhoe M, Hikosaka O, Rangel A (2014). Attention, reward, and information seeking. Journal of Neuroscience.

[CR26] Gottlieb J, Oudeyer P-Y (2018). Towards a neuroscience of active sampling and curiosity. Nature Reviews Neuroscience.

[CR27] Hansen HA, Irons JL, Leber AB (2019). Taking stock: The role of environmental appraisal in the strategic use of attentional control. Attention, Perception, and Psychophysics.

[CR28] Hayhoe MM (2017). Vision and Action. Annual Review of Vision Science.

[CR29] Hills TT, Todd PM, Goldstone RL (2010). The central executive as a search process: Priming exploration and exploitation across domains. Journal of Experimental Psychology: General.

[CR30] Hoppe D, Rothkopf CA (2019). Multi-step planning of eye movements in visual search. Scientific Reports.

[CR31] Horan M, Daddaoua N, Gottlieb J (2019). Parietal neurons encode information sampling based on decision uncertainty. Nature Neuroscience.

[CR32] Irons JL, Leber AB (2016). Choosing attentional control settings in a dynamically changing environment. Attention, Perception, and Psychophysics.

[CR33] Irons JL, Leber AB (2018). Characterizing individual variation in the strategic use of attentional control. Journal of Experimental Psychology: Human Perception and Performance.

[CR34] Irons JL, Leber AB (2020). Developing an individual profile of attentional control strategy. Current Directions in Psychological Science.

[CR35] Itti L, Koch C (2000). A saliency-based search mechanism for overt and covert shifts of visual attention. Vision Research.

[CR36] Jarvstad A, Rushton SK, Warren PA, Hahn U (2012). Knowing when to move on: Cognitive and perceptual decisions in time. Psychological Science.

[CR37] Kaptein NA, Theeuwes J, van der Heijden AHC (1995). Search for a conjunctively defined target can be selectively limited to a color-defined subset of elements. Journal of Experimental Psychology: Human Perception and Performance.

[CR38] Kristjánsson Á, Jóhannesson ÓI, Thornton IM (2014). Common attentional constraints in visual foraging. PLoS ONE.

[CR39] Kümmerer, M., Wallis, T. S. A., & Bethge, M. (2016). *DeepGaze II: Reading fixations from deep features trained on object recognition*. http://arxiv.org/abs/1610.01563

[CR40] Lisi M, Morgan MJ, Solomon JA (2022). Perceptual decisions and oculomotor responses rely on temporally distinct streams of evidence. Communications Biology.

[CR41] Liston DB, Stone LS (2008). Effects of prior information and reward on oculomotor and perceptual choices. Journal of Neuroscience.

[CR42] Lleras, A., Buetti, S., & Xu, Z. J. (2022). Incorporating the properties of peripheral vision into theories of visual search. *Nature Reviews Psychology*, 1–15. 10.1038/s44159-022-00097-1

[CR43] Morvan C, Maloney LT (2012). Human visual search does not maximize the post-saccadic probability of identifying targets. PLoS Computational Biology.

[CR44] Najemnik J, Geisler WS (2005). Optimal eye movement strategies in visual search. Nature.

[CR45] Najemnik J, Geisler WS (2008). Eye movement statistics in humans are consistent with an optimal search strategy. Journal of Vision.

[CR46] Nakayama K, Martini P (2011). Situating visual search. Vision Research.

[CR47] Näsänen R, Ojanpää H, Kojo I (2001). Effect of stimulus contrast on performance and eye movements in visual search. Vision Research.

[CR48] Navalpakkam V, Koch C, Rangel A, Perona P (2010). Optimal reward harvesting in complex perceptual environments. Proceedings of the National Academy of Sciences.

[CR49] Ng GJP, Lleras A, Buetti S (2018). Fixed-target efficient search has logarithmic efficiency with and without eye movements. Attention, Perception, and Psychophysics.

[CR50] Nowakowska A, Clarke ADF, Hunt AR (2017). Human visual search behaviour is far from ideal. Proceedings of the Royal Society B: Biological Sciences.

[CR51] Nowakowska A, Clarke ADF, von Seth J, Hunt AR (2021). Search strategies improve with practice, but not with time pressure or financial incentives. Journal of Experimental Psychology. Human Perception and Performance.

[CR52] Paeye C, Schütz AC, Gegenfurtner KR (2016). Visual reinforcement shapes eye movements in visual search. Journal of Vision.

[CR53] Paulun VC, Schütz AC, Michel MM, Geisler WS, Gegenfurtner KR (2015). Visual search under scotopic lighting conditions. Vision Research.

[CR54] Peterson MF, Eckstein MP (2012). Looking just below the eyes is optimal across face recognition tasks. Proceedings of the National Academy of Sciences.

[CR55] Petitet P, Attaallah B, Manohar SG, Husain M (2021). The computational cost of active information sampling before decision-making under uncertainty. Nature Human Behaviour.

[CR56] Pomplun M, Garaas TW, Carrasco M (2013). The effects of task difficulty on visual search strategy in virtual 3D displays. Journal of Vision.

[CR57] Renninger LW, Verghese P, Coughlan J (2007). Where to look next? Eye movements reduce local uncertainty. Journal of Vision.

[CR58] Resulaj A, Kiani R, Wolpert DM, Shadlen MN (2009). Changes of mind in decision-making. Nature.

[CR59] Rosenholtz R (2016). Capabilities and limitations of peripheral vision. Annual Review of Vision Science.

[CR60] Schütz AC, Braun DI, Gegenfurtner KR (2011). Eye movements and perception: A selective review. Journal of Vision.

[CR61] Schütz AC, Trommershäuser J, Gegenfurtner KR (2012). Dynamic integration of information about salience and value for saccadic eye movements. Proceedings of the National Academy of Sciences.

[CR62] Shen J, Reingold EM, Pomplun M (2000). Distractor ratio influences patterns of eye movements during visual search. Perception.

[CR63] Shenhav A, Musslick S, Lieder F, Kool W, Griffiths TL, Cohen JD, Botvinick MM (2017). Toward a Rational and Mechanistic Account of Mental Effort. Annual Review of Neuroscience.

[CR64] Smith PL, Little DR (2018). In defense of the small-N design. Psychonomic Bulletin and Review.

[CR65] Sobel KV, Cave KR (2002). Roles of salience and strategy in conjunction search. Journal of Experimental Psychology: Human Perception and Performance.

[CR66] Stewart EEM, Ludwig CJH, Schütz AC (2022). Humans represent the precision and utility of information acquired across fixations. Scientific Reports.

[CR67] Stewart EEM, Valsecchi M, Schütz AC (2020). A review of interactions between peripheral and foveal vision. Journal of Vision.

[CR68] Strasburger H, Rentschler I, Jüttner M (2011). Peripheral vision and pattern recognition: A review. Journal of Vision.

[CR69] Stritzke M, Trommershäuser J, Gegenfurtner KR (2009). Effects of salience and reward information during saccadic decisions under risk. Journal of the Optical Society of America A.

[CR70] Tatler BW, Hayhoe MM, Land MF, Ballard DH (2011). Eye guidance in natural vision: reinterpreting salience. Journal of Vision.

[CR71] Thaler L, Schütz AC, Goodale MA, Gegenfurtner KR (2013). What is the best fixation target? The effect of target shape on stability of fixational eye movements. Vision Research.

[CR72] Tsank Y, Eckstein MP (2017). Domain specificity of oculomotor learning after changes in sensory processing. Journal of Neuroscience.

[CR73] Vanunu Y, Hotaling JM, Le Pelley ME, Newell BR (2021). How top-down and bottom-up attention modulate risky choice. Proceedings of the National Academy of Sciences.

[CR74] Verghese P (2012). Active search for multiple targets is inefficient. Vision Research.

[CR75] Williams CC, Pollatsek A (2007). Searching for an O in an array of Cs: Eye movements track moment-to-moment processing in visual search. Perception and Psychophysics.

[CR76] Wolf C, Lappe M (2020). Top-down control of saccades requires inhibition of suddenly appearing stimuli. Attention, Perception, and Psychophysics.

[CR77] Wolf C, Wagner I, Schütz AC (2019). Competition between salience and informational value for saccade adaptation. Journal of Vision.

[CR78] Wolfe JM (2021). Guided Search 6.0: An updated model of visual search. Psychonomic Bulletin & Review.

[CR79] Yang SCH, Lengyel M, Wolpert DM (2016). Active sensing in the categorization of visual patterns. ELife.

[CR80] Zhou Y, Yu Y (2021). Human visual search follows a suboptimal Bayesian strategy revealed by a spatiotemporal computational model and experiment. Communications Biology.

