# Supplementary material for: Humans trade off search costs and accuracy in a combined visual search and perceptual task
Source: Atten Percept Psychophys. Author manuscript; Available in PMC 2023 Jan 26. (PMC9816200; doi:10.3758/s13414-022-02600-5)
Supplement: Supplementary Material [file EMS158698-supplement-Supplementary_Material.docx]

**Humans trade-off search costs and accuracy in a combined visual search and perceptual task**

**– Supplementary Information –**

Ilja Wagner, Dion Henare, Jan Tünnermann, Anna Schubö & Alexander C. Schütz

# Supplementary methods

*Ideal observer prediction.* We hypothesized that the target which a participant chooses to search and discriminate in the double-target condition depends on both the probability of correctly discriminating the target and the search costs to find the target amongst same-colored distractors. Optimally trading off those two factors should enable participants to optimize how many targets they can correctly discriminate throughout the double-target condition (i.e., their monetary gain per unit of time) and, by this, maximize their accumulated bonus payout. To determine the expected value of available target options, we used data from the single-target condition and calculated the prospective monetary gains per unit of time of available target options, separately for each set size and participant. For this, we used information about how well each individual participant in the single-target condition could discriminate easy and difficult targets (proportion correct, pc*_E_* or pc*_D_*), how many points could be gained (gain) and lost (loss) by discriminating a target correctly or incorrectly and how much time individual participants in the single-target condition spent, on average, per trial. The time spent per trial was calculated for each target separately and was broken down into three components: the time it took to plan search at trial start (planning time, pt*_E_* or pt*_D_*), the average time spent in AOIs around elements during search (inspection time, it*_E_* or it*_D_*; see Calculating planning, inspection and response time for how inspection time was calculated) and the time spent to give the response after the target was found (response time, rt*_E_* or rt*_D_*). Based on this, we estimated the relative expected value of target options [$\Delta ev(N_{E,}N_{D})$] under different numbers of same-colored elements ($N_{E}$ or $N_{D}$), with *N_D_* corresponding to 10 − *N_E_*:

$\mathrm{ev}_{E}(N_{E})=\frac{\mathrm{pc}_{E}\cdot gain+\left( 1-\mathrm{pc}_{E} \right)\cdot loss}{\frac{N_{E}-1}{2}\cdot\mathrm{it}_{E}+\mathrm{pt}_{E}+\mathrm{rt}_{E}}$ (1)

$\mathrm{ev}_{D}(N_{D})=\frac{\mathrm{pc}_{D}\cdot gain+\left( 1-\mathrm{pc}_{D} \right)\cdot loss}{\frac{N_{D}-1}{2}\cdot\mathrm{it}_{D}+\mathrm{pt}_{D}+\mathrm{rt}_{D}}$ (2)

$\Delta ev(N_{E,}N_{D})=\mathrm{ev}_{D}(N_{D})-\mathrm{ev}_{E}(N_{E})$ (3)

Here, a target’s expected value is defined as the ratio between the probability to discriminate a target correctly, and thus, earn the associated monetary reward, and the required time to find and discriminate the target among same-colored distractors (i.e., the target’s search costs). We assume that participants will act like an ideal observer, who decides at trial start which target to search for and that will only inspect elements from the set of the chosen target, while searching for the chosen target. Since targets and distractors can only be discriminated with foveal vision, an ideal observer will need to fixate, on average, half of the distractors from the set of the chosen target before finding the target. Furthermore, since the ideal observer acts optimally, factors such missing a target (i.e., fixating it but still continuing search) or fixating the same stimulus multiple times are explicitly neglected, since they result in suboptimal behavior. Using $\Delta ev(N_{E},N_{D})$, we can infer the probability *P* to choose an easy target T*_E_* under different relative set size conditions [$P\left( T_{E}|N_{E},N_{D} \right)$]. Under a winner-take-all decision strategy, participants should always choose to discriminate easy targets, when Equation 3 becomes negative for a given set size condition. Conversely, when Equation 3 becomes positive, participants should always choose to discriminate difficult targets whenever the corresponding set size condition is shown. Finally, whenever Equation 3 is zero, participants should favor both targets equally, since none has a clearly superior expected value

$P\left( T_{E}|N_{E},N_{D} \right)=\left\{ \begin{aligned} 1 \mathrm{if} \Delta ev(N_{E},N_{D})<0 \\ 0.50 \mathrm{if} \Delta ev(N_{E},N_{D})=0 \\ 0 \mathrm{if} \Delta ev(N_{E},N_{D})>0 \end{aligned} \right.$ (4)

Weighting the result of Equation 4 with the gain vectors of available target options (Equations 1 and 2), taking the sum of the weighted gains and averaging over the resulting vector yields the theoretically possible monetary gain per unit of time a participant could achieve if consistently discriminating higher-gain targets and perfectly restricting visual search to elements from the set of the chosen target

$\mathrm{perf}_{\mathrm{opt}}=\frac{1}{9}\sum_{N_{E}=1}^{9} \left( P\left( T_{E}|N_{E},N_{D} \right)\cdot\mathrm{ev}_{E}(N_{E}) \right)+(\left( 1-P\left( T_{E}|N_{E},N_{D} \right) \right)\cdot\mathrm{ev}_{D}(N_{D}))$ (5)

*Adding noise at the decision and fixation level.* Equations 1–5 follow two fairly strict assumptions about participants’ behavior in our task: First, an ideal observer (and thus, participants) can perfectly estimate which target will yield a higher gain in trials and always selects the set with the higher-gain target as its chosen set. This assumption also implies that participants have a chosen set at all, and are not just searching randomly through elements of both sets. Second, the ideal observer can perfectly restrict fixations during search to elements from the set of the chosen target (which, under assumption one, is the higher-gain target). However, both assumptions are not necessarily reflected in the empirical data (Figure 4 and Supplementary figure S 2, Figure 5). To relax them, we built a model that allows for some degree of noise in decision-making and in choices for which elements to fixate. For this, our model, first, adds variable decision noise to the relative gain estimates of participants to reflect occasional preferences for lower-gain targets. In a second step, independent noise is introduced at the fixation level, relaxing the fixation bias to elements from the set of the higher-gain target and leading to some proportion of random fixations on elements from the set of the lower-gain target.

To obtain noisy gain estimates, Gaussian noise was injected into relative gain estimates from Equation 3. For this, noise samples were drawn from a Gaussian distribution with $\mu$ = 0 and $\sigma$ as free parameter, and then summed with relative gain estimates from Equation 3 (Figure 2A). This procedure was repeated 100,000 times and the result of this was averaged to obtain the final noisy gain estimates. In contrast to noise at the fixation level, the free parameter at the decision level controls the magnitude by which individual relative gain estimates are corrupted by decision noise: For example, a free parameter of zero corresponds to a situation where participants can perfectly estimate the relative gain of available target options (i.e., they can perfectly estimate which target has the higher gain, given their individual performance from the single-target condition; see Equations 1–3) and the larger the free parameter becomes the stronger individual gain estimates deviate from the theoretical optimum. Critically, unlike noise at the fixation level, those deviations might lead to occasional choices for lower-gain targets if the injected decision noise is large enough.

The noisy fixation bias was obtained by transforming noisy gain estimates (i.e., after decision noise was added) to a cumulative Gaussian distribution function with $\mu$ = 0 and $\sigma$ as free parameter; Since the results of this transformation can range between 0 and 1, we multiplied the transformation results by two, so that they are in the desired value range for the fixation bias parameter *b* (see formal description of model):

$b(N_{E,}N_{D})=\Phi\left( \frac{\Delta ev(N_{E,}N_{D})-\mu}{\sigma} \right)\cdot2=\frac{1}{2}\left[ 1+\mathrm{erf}\left( \frac{\Delta ev(N_{E,}N_{D})-\mu}{\sigma\sqrt{2}} \right)\cdot2 \right]$ (6)

Thus, here, the free parameter controls the magnitude by which elements from the lower-gain set are fixated during search: For example, if the free parameter is zero, only elements from the higher-gain set are fixated during search [i.e., $b(N_{E,}N_{D})$ is either zero or two, depending on $\Delta ev(N_{E,}N_{D})$] (Figure 2A, rightmost plot in the illustration). The larger the free parameter becomes the more random fixations to elements from the lower-gain set are made, until elements from both sets are favored equally. Critically, adding noise at the fixation level thus only influences the degree to which participants are predicted to restrict fixations to elements from the set of the higher-gain target. It does, however, not influence the gain estimates of available target options, and thus, which target participants predict to have the higher gain.

Choosing fixation locations in our paradigm can be considered a stochastic problem of biased selection from two urns without replacement (see also Wallenius, 1963). Here, one urn contained all elements from the easy set (target + the number of easy distractors in a given set size condition), whereas the other urn contained all elements from the difficult set. At each fixation, participants first decided which urn they want to sample from (i.e., if they want to fixate an element from the easy or difficult set), and second, drew an element from the chosen urn (i.e., made a gaze shift that either landed on a target or distractor). The preference to sample from one or the other urn was expressed via the fixation bias *b*. Hence, the model was implemented as a binary decision tree, which was traversed recursively (Figure 2). At each recursion step, we first calculated the biased probability to either fixate an element from the easy or difficult set (i.e., the probability to draw an element from one or the other urn), and second, the probability to fixate either a distractor or target from the corresponding set. When a distractor was fixated, one element from the respective stimulus set was removed and the next recursion step was initiated. When a target was fixated, recursion was terminated and all probabilities along the tree path, leading to the target, were multiplied to obtain the probability of this outcome. After traversing all nodes in a tree, the probabilities of all paths leading to the easy or the difficult target were summed to obtain the overall probability of choosing the easy or the difficult target. Furthermore, the fixations on the set of the chosen and the set of the non-chosen target were summed to calculate how many elements from both sets were fixated on average before a target was found.

Formally, at the first recursion step, we initialized the decision tree by setting the cumulative probability *P* that a target T was not found up to this recursion step [$P\left( \bar{T}_{0} \right)$] to one. This probability was then updated at each recursion step *i* by calculating the product between the probability of a fixation F to an element from either the easy set *E* or the difficult set *D*, the probability that a distractor $\bar{T}$ from set *E* or *D* will be fixated and the cumulative probability that a target was not found up to the previous recursion step *i* − 1:

$P\left( \bar{T}_{[1 .. i]} \right)=P\left( F_{Ei} \right)\cdot P\left( \bar{T}_{Ei}|F_{Ei} \right)\cdot P\left( \bar{T}_{i-1} \right)$ (7)

$P\left( \bar{T}_{[1 .. i]} \right)=P\left( F_{Di} \right)\cdot P\left( \bar{T}_{Di}|F_{Di} \right)\cdot P\left( \bar{T}_{i-1} \right)$ (8)

At each recursion step *i*, the probability of fixating an element from either set *E* or *D* was calculated as the ratio between the number of remaining elements in the corresponding set (*N_Ei_* or *N_Di_*) and the number of all remaining elements. Since there are only two sets in our paradigm, the probability to fixate an element from set *D* can be expressed as the remainder of the probability to fixate an element from set *E*:

$P\left( F_{Ei} \right)=\frac{N_{Ei}}{N_{Ei}+N_{Di}}$ (9)

$P\left( F_{Di} \right)=1-P\left( F_{Ei} \right)$ (10)

In order to bias fixations to elements from one or the other set, we introduced a bias parameter *b*, whose value represents the preference of participants to fixate elements of set *E* or *D* by increasing or decreasing the relative set sizes. Here, $b\mathbb{\in R :}0 \leq b \leq2$, with values < 1 corresponding to a preference to fixate elements from set *E* during search, values > 1 corresponding to a preference to fixate elements from set *D* and unity corresponding to no bias. Values of zero or two indicate extreme fixation biases where only elements from set *E* (*b* = 0) or set *D* (*b* = 2) are fixated. This replaces Equations 9 and 10 with:

$P\left( F_{Ei} \right)=\left\{ \begin{aligned} \frac{N_{Ei}}{N_{Ei}+{b\cdot N}_{Di}}\mathrm{when}b<1 \\ \frac{{\left( 2-b \right)\cdot N}_{Ei}}{{\left( 2-b \right)\cdot N}_{Ei}+N_{Di}}\mathrm{when}b\geq1 \end{aligned} \right.$ (11)

$P\left( F_{Di} \right)=\left\{ \begin{aligned} \frac{b\cdot N_{Di}}{N_{Ei}+{b\cdot N}_{Di}}\mathrm{when}b<1 \\ \frac{N_{Di}}{{\left( 2-b \right)\cdot N}_{Ei}+N_{Di}}\mathrm{when}b\geq1 \end{aligned} \right.$ (12)

Since each stimulus set always contained exactly one target, we, next, calculated the probability to fixate (and thus find) a target T in the selected set at recursion step *i* as the inverse of the respective number of remaining set-specific elements:

$P\left( T_{Ei}|F_{Ei} \right)=\frac{1}{N_{Ei}}$ (13)

$P\left( T_{Di}|F_{Di} \right)=\frac{1}{N_{Di}}$ (14)

Similarly, the probability to fixate (and thus find) a distractor $\bar{T}$ in the selected set at recursion step *i* can be calculated as the remainder of the probability to fixate a target:

$P\left( \bar{T}_{Ei}|F_{Ei} \right)=1-P\left( T_{Ei}|F_{Ei} \right)$ (15)

$P\left( \bar{T}_{Di}|F_{Di} \right)=1-P\left( T_{Di}|F_{Di} \right)$ (16)

The product of Equations 11 and 12, Equations 13 and 14 and Equations 7 and 8 yields the probability of finding target T in the easy or difficult set at recursion step *i*:

$P\left( {T_{Ei}\cap F}_{Ei} \right)=P\left( F_{Ei} \right)\cdot P\left( T_{Ei}|F_{Ei} \right)\cdot P\left( \bar{T}_{i-1} \right)$ (17)

$P\left( {T_{Di}\cap F}_{Di} \right)=P\left( F_{Di} \right)\cdot P\left( T_{Di}|F_{Di} \right)\cdot P\left( \bar{T}_{i-1} \right)$ (18)

For each recursion step *i*, multiple outcomes *j*, that lead to a target from sets *E* or *D* being fixated, might exist (see, for example, second fixation in Figure 2B). Those outcomes (hits *H*) can be treated as parts of two sets, containing the probabilities of all possible outcomes across the entire decision tree that led to either a target from set *E* or set *D* being found:


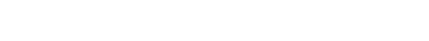

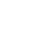


$H_{E}=\left\{ P\left( {T_{E1}\cap F}_{E1} \right)\ldots P\left( {T_{Ej}\cap F}_{Ej} \right) \right\}$ (19)

$H_{D}=\left\{ P\left( {T_{D1}\cap F}_{D1} \right)\ldots P\left( {T_{Dj}\cap F}_{Dj} \right) \right\}$ (20)

To obtain predictions for the probabilities that participants will either discriminate the easy or difficult target for a given set size condition we took the sum over all probabilities in the two sets *H_E_* and *H_D_*:

$P\left( T_{E}|N_{E},N_{D} \right)=\sum_{k=1}^{j} H_{Ek}$ (21)

$P\left( T_{D}|N_{E},N_{D} \right)=\sum_{k=1}^{j} H_{Dk}$ (22)

For each outcome *j* that led to a target *E* or *D* being fixated we can, additionally, calculate the number of required fixations *RF* on elements from sets *E* and *D* until a target from sets *E* or *D* was found as the difference between the initial number of set elements at the root node and the number of remaining set elements when the target was found at outcome *j*

${RF}_{E[1.. j]}=\{N_{E}-N_{E1}\ldots N_{E}-N_{Ej}\}$ (23)

${RF}_{D[1.. j]}=\{N_{D}-N_{D1}\ldots N_{D}-N_{Dj}\}$ (24)

To obtain predictions about how many elements from the set of the chosen (F_CS_) and not-chosen (F_NCS_) target will be fixated on average before the respective target is found at a given set size condition, we took the sum of the products between Equations 19 and 20 and Equations 23 and 24:

$F_{\mathrm{CS}}=\sum_{k=1}^{j} \left[ H_{Ek}\cdot{RF}_{Ek} \right]+[H_{Dk}\cdot{RF}_{Dk}]$ (25)

$F_{\mathrm{NCS}}=\sum_{k=1}^{j} \left[ H_{Ek}\cdot{RF}_{DK} \right]+[H_{Dk}\cdot{RF}_{EK}]$ (26)

Finally, using the predictions from Equations 21 and 22 and Equations 25 and 26 we can extend Equation 5 and directly predict the overall average monetary gain per unit of time over all set size conditions, participants are predicted to have, given a certain fixation bias and decision noise:


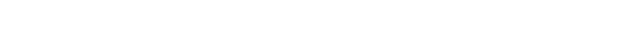


$\mathrm{payout}_{E}(N_{E},N_{D})=\left[ \mathrm{pc}_{E}\cdot\mathrm{gain}\cdot P\left( T_{E}|N_{E},N_{D} \right) \right]+\left[ \left( 1-\mathrm{pc}_{E} \right)\cdot\mathrm{loss}\cdot P\left( T_{E}|N_{E},N_{D} \right) \right]$ (27)

$\mathrm{payout}_{D}(N_{E},N_{D})=\left[ \mathrm{pc}_{D}\cdot\mathrm{gain}\cdot P\left( T_{D}|N_{E},N_{D} \right) \right]+\left[ \left( 1-\mathrm{pc}_{D} \right)\cdot\mathrm{loss}\cdot P\left( T_{D}|N_{E},N_{D} \right) \right]$ (28)

$\mathrm{searchTime}_{E}(N_{E},N_{D})=\left[ \left( F_{\mathrm{CS}}+F_{\mathrm{NCS}}-1 \right)\cdot\left( P\left( T_{E}|N_{E},N_{D} \right)\cdot\mathrm{it}_{E} \right)+\left( P\left( T_{E}|N_{E},N_{D} \right)\cdot\mathrm{pt}_{E} \right)+\left( P\left( T_{E}|N_{E},N_{D} \right)\cdot\mathrm{rt}_{E} \right) \right]$ (29)

$\mathrm{searchTime}_{D}(N_{E},N_{D})=\left[ \left( F_{\mathrm{CS}}+F_{\mathrm{NCS}}-1 \right)\cdot\left( P\left( T_{D}|N_{E},N_{D} \right)\cdot\mathrm{it}_{D} \right)+\left( P\left( T_{D}|N_{E},N_{D} \right)\cdot\mathrm{pt}_{D} \right)+\left( P\left( T_{D}|N_{E},N_{D} \right)\cdot\mathrm{rt}_{D} \right) \right]$ (30)

$\mathrm{perf}_{\mathrm{pred}}=\frac{1}{9}\sum_{N_{E}=1}^{9} \frac{\mathrm{payout}_{E}(N_{E},N_{D})+\mathrm{payout}_{D}(N_{E},N_{D})}{\mathrm{searchTime}_{E}(N_{E},N_{D})+\mathrm{searchTime}_{D}(N_{E},N_{D})}$ (31)

Since our model predicts the number of fixations until a target is found (i.e., it includes a fixation on the target) the predictions in Equations 29 and 30 have to be corrected by one fixation, to be in line with how we calculate inspection time (i.e., without considering the target fixation before response; for details, see Calculating planning, inspection and response time).

To evaluate fits, empirical proportion choices for easy targets and empirical proportion fixations on elements from the chosen set, each separately for the nine different set size conditions, were passed into the loss function. We used MatLab’s fminsearch algorithm to minimize the sum of squared residuals between model (RSS) predictions and the empirical data. To be in line with proportion fixations on elements from the chosen set, as predicted by the model, empirical proportion fixations, as passed into the loss function, were calculated by considering (if available) the last fixation participants made in trials before placing a response (which, in most cases, landed on a target). Since it is not strictly part of the ongoing search, this last fixation was omitted when calculating proportion gaze shifts in Figure 5, and thus, the proportion gaze shifts were calculated slightly differently when passed into the loss function of the model.

# Supplementary figures


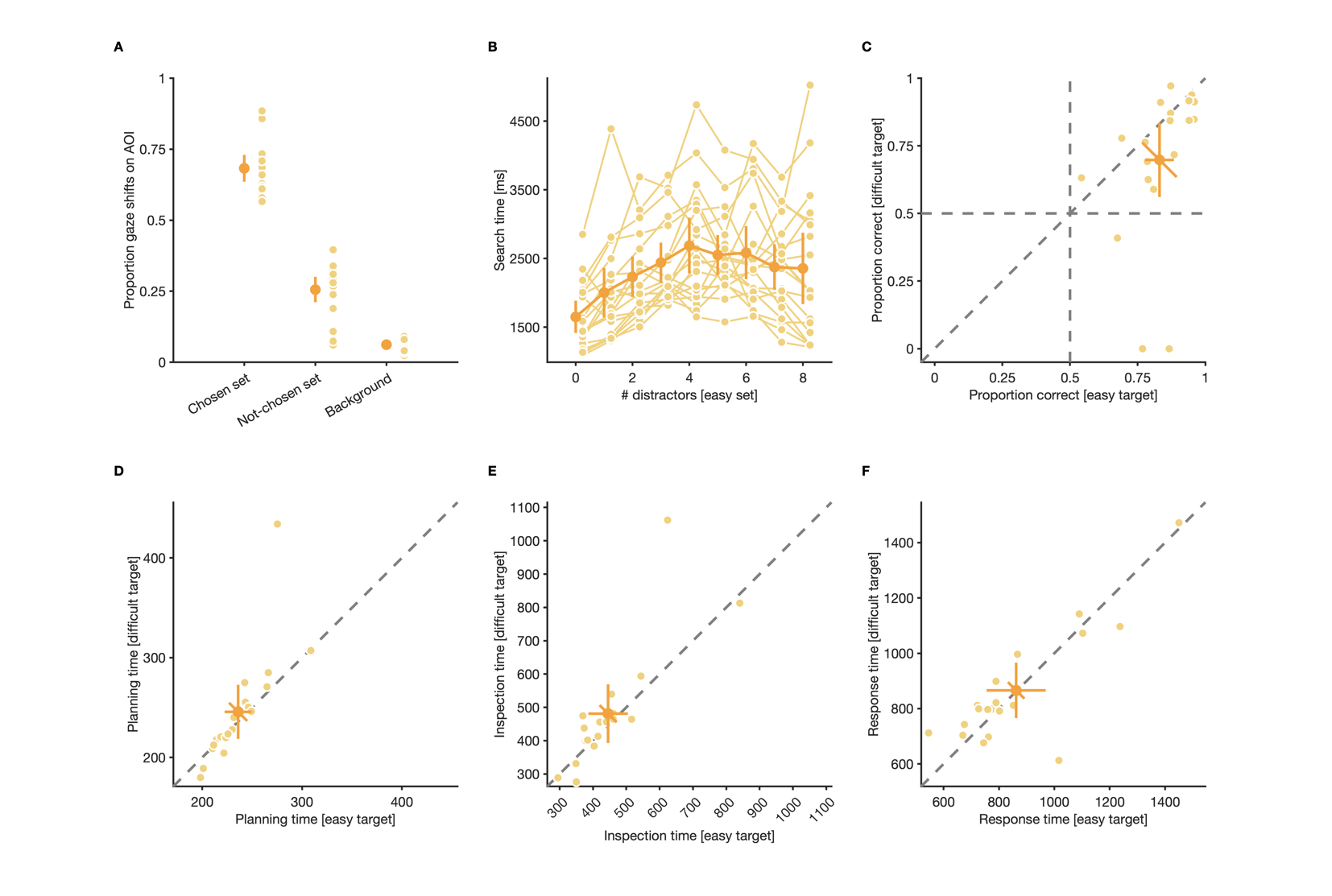


Supplementary figure S1. Search behavior and perceptual performance in the double-target condition. (A) Proportion gaze shifts that landed within areas of interest (AOIs). (B) Search time (average time between onset of stimuli and response) for different distractor numbers. Search times for easy and difficult targets were analyzed together. (C) Discrimination performance, (D) planning time (time between stimulus array onset and offset of the first gaze shift in a trial), (E) inspection time (average time between entering and leaving AOIs around stimuli) and (F) response time (time between offset of the last gaze shift in a trial and response) for easy and difficult targets. (A)–(F) Small, light dots are data from individual participants, large, dark dots are means across participants. Error bars are 95% confidence intervals.


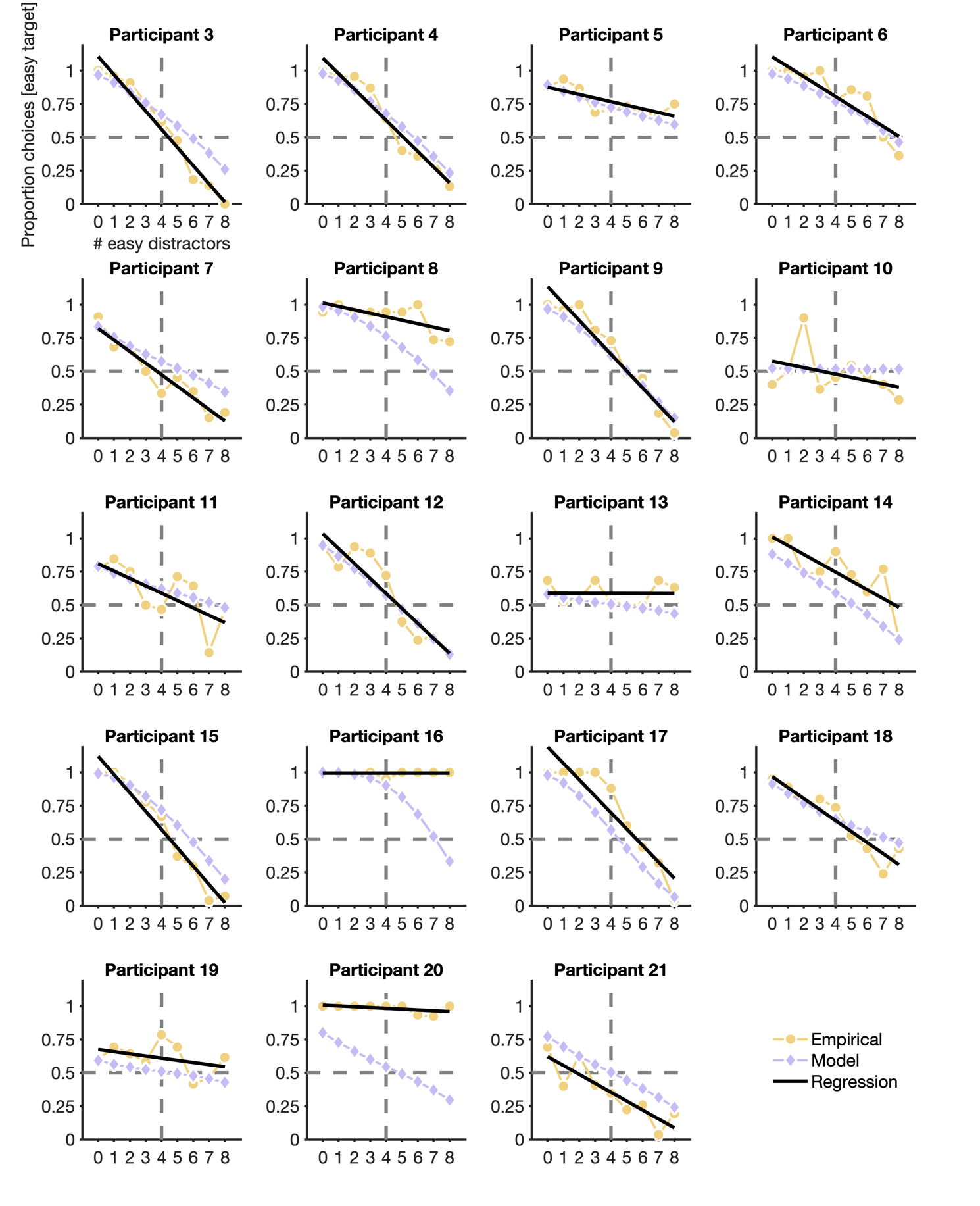


Supplementary figure S 2. Empirical and predicted probability to choose easy targets for different set size conditions in the double-target condition. Small, light dots are empirical (orange) and predicted (purple) mean proportions for individual set size conditions (number easy distractors in a trial) and black lines are fits of linear regressions.


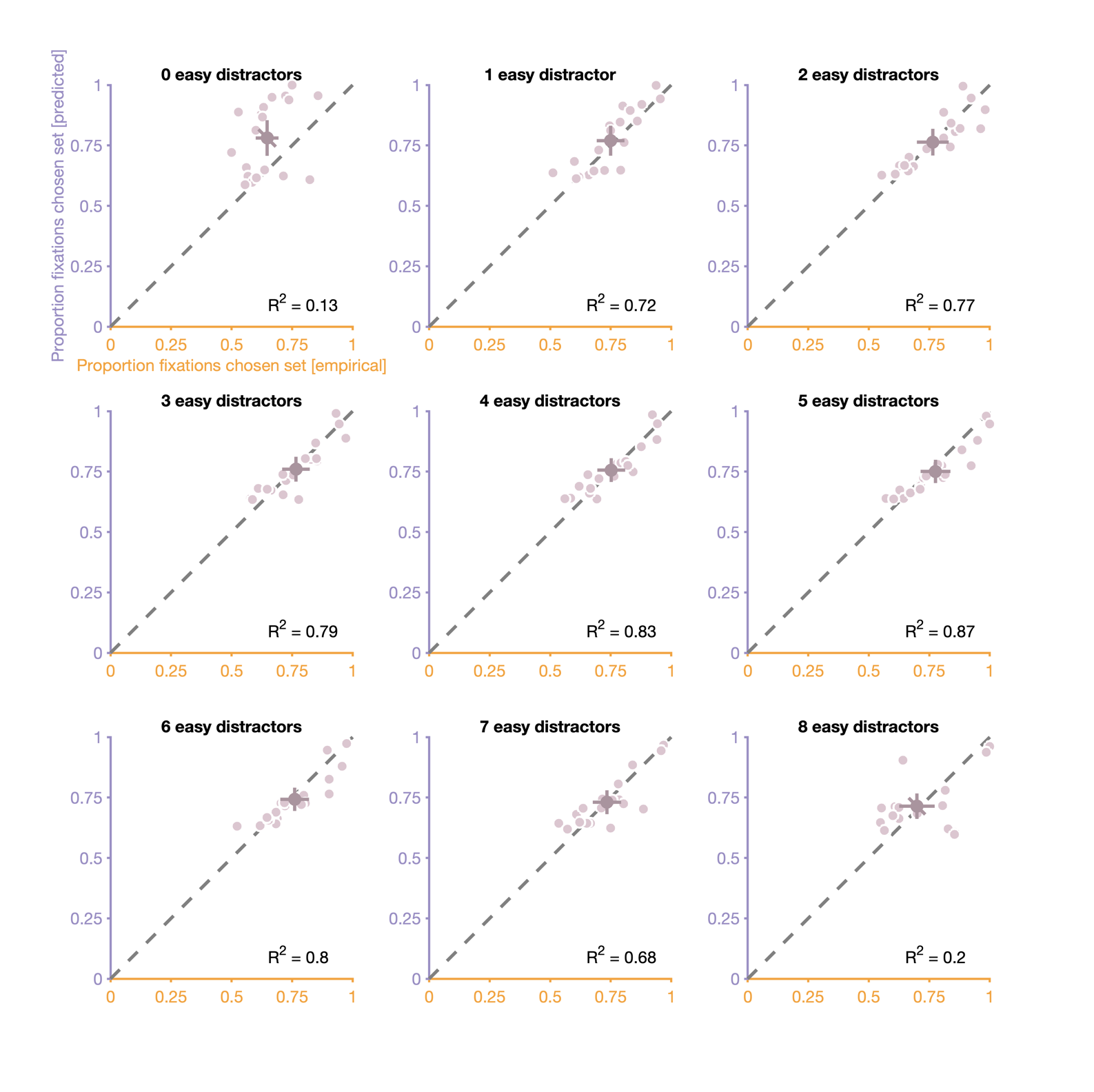


Supplementary figure S 3. Empirical and predicted proportions gaze shifts on elements from the chosen set for different set size conditions. Small, light dots are mean proportions from individual participants, large, dark dots are the mean proportions across participants. Error bars are 95% confidence intervals.

# References

Wallenius, K. T. (1963). *Biased Sampling: The Noncentral Hypergeometric Probability Distribution*. Doctoral dissertation, Stanford University.
